# Iron-Based High-Temperature Alloys: Alloying Strategies and New Opportunities

**DOI:** 10.3390/ma18132989

**Published:** 2025-06-24

**Authors:** Yingjie Qiao, Yanzhao Ni, Kun Yang, Peng Wang, Xiaodong Wang, Ruiliang Liu, Bin Sun, Chengying Bai

**Affiliations:** 1 Key Laboratory of Superlight Materials and Surface Technology, Ministry of Education, College of Materials Science and Chemical Engineering, Harbin Engineering University, Harbin 150001, China; qiaoyingjie@hrbeu.edu.cn (Y.Q.); wangxiaodong@hrbeu.edu.cn (X.W.); liuruiliang@hrbeu.edu.cn (R.L.); sunbin2040@163.com (B.S.); 2 College of Materials Science and Technology, Nanjing University of Aeronautic and Astronautics, Nanjing 210016, China; kun.yang@nuaa.edu.cn; 3 School of Materials Science and Engineering, China University of Petroleum (East China), Qingdao 266580, China; 20230026@upc.edu.cn

**Keywords:** Fe-based alloy, alloying strategy, high-entropy alloy, additive manufacturing, high-temperature application

## Abstract

Iron-based high-temperature alloys are engineered to withstand extreme conditions, including elevated temperatures, mechanical stress, and corrosive environments. These alloys play a critical role in industries such as aerospace, power generation, and chemical processing, where materials must maintain structural integrity and performance under demanding operational conditions. This review examines recent advancements in iron-based alloys, with a focus on alloying strategies, high-temperature performance, and applications. Traditional approaches—including alloy design, microstructure control, process optimization, and computational modeling—continue to enhance alloy performance. Furthermore, emerging technologies such as high-entropy alloy (HEA) design, additive manufacturing (AM), nanostructured design with nanophase strengthening, and machine learning/artificial intelligence (ML/AI) are revolutionizing the development of iron-based superalloys, creating new opportunities for advanced material applications.

## 1. Introduction

Advanced high-temperature materials encompass several key categories: high-temperature alloys (HTAs, also known as superalloys) [1,2], refractory metals [3], ceramics, intermetallic compounds, metal matrix composites, ceramic matrix composites, C/C composites, and gradient functional materials [4,5]. These materials are critical for industries operating under extreme conditions, including aerospace, automotive, power generation, and industrial manufacturing [6,7,8]. High-temperature (HT) materials are specifically engineered to endure mechanical stress, thermal cycling, oxidation, and corrosion at elevated temperatures [9,10]. Among these, HTAs have become the most widely adopted due to their exceptional thermal stability, good machinability, outstanding mechanical properties, and superior resistance to oxidation and corrosion at high temperatures [1,2,8,11]. Designed to maintain mechanical strength and structural stability at temperatures typically above 600 °C, HTAs are crucial for extreme environments where conventional materials would fail under intense heat, pressure, or chemical exposure [12]. These alloys are primarily based on nickel (Ni) [13], cobalt (Co) [14], iron (Fe) [11], or titanium (Ti) [15], and are frequently alloyed with chromium (Cr), molybdenum (Mo), and aluminum (Al) to enhance their thermal and mechanical properties.

**Figure 1:**
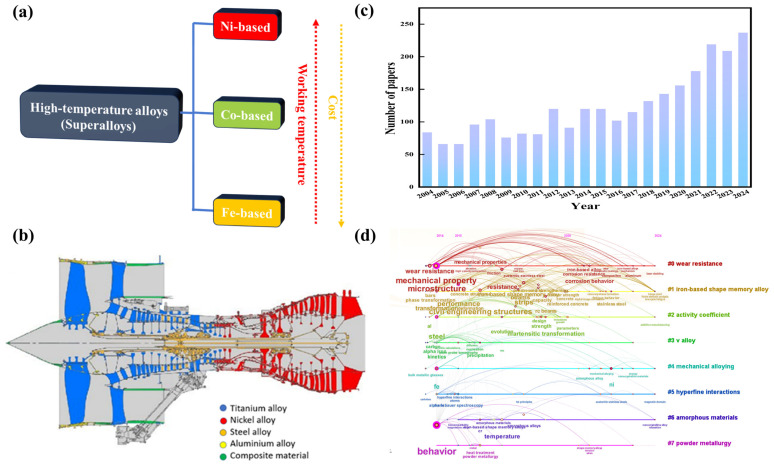
(**a**) Typical classification of HTAs. (**b**) Main materials used for the Trent 800 engine manufacturing. Reprinted with permission from Ref. [18]. Copyright 2023, Springer Nature (**c**,**d**) The trend in the number of publications (last 20 years) and variation in the keywords burst detection analysis (last 10 years) from VOSviewer 1.6.20 as part of the topic “Iron-based alloys” from Web of science.

HTAs are classified into three primary categories according to their dominant metallic base: Fe-based, Co-based, and Ni-based superalloys (Figure 1a) [16,17,18]. Among these, Ni-based alloys currently represent the most extensively utilized category [16], prized for their exceptional heat resistance and oxidation stability, which make them indispensable for aerospace (Figure 1b) and power generation applications [19]. Their remarkable strength primarily derives from the formation of a protective oxide layer that effectively prevents further material degradation.

Co-based alloys demonstrate superior performance in high-wear environments and are commonly employed in turbine blade applications [14]. In comparison, Fe-based alloys offer a cost-effective alternative [20], and are typically employed in less extreme conditions than nickel or cobalt alloys while still maintaining considerable heat resistance.

The selection of HTAs is governed by application-specific requirements including operating temperature ranges, mechanical stress conditions, and environmental exposure. A critical consideration involves balancing performance requirements with cost-effectiveness. Among the available options, Fe-based HTAs distinguish themselves through compelling advantages, most notably their exceptional cost efficiency. Given the natural abundance of Fe and its significantly lower cost compared to nickel, cobalt, or titanium, Fe-based alloys emerge as the preferred solution for large-scale industrial applications [17,21,22,23,24,25,26,27,28,29], where material costs substantially influence technological and economic feasibility. Beyond their economic advantages, Fe-based HTAs deliver competitive HT performance, exhibiting excellent strength retention, creep resistance, and fabrication ease [21,22]. These alloys demonstrate superior manufacturability, including enhanced weldability [30,31,32], casting characteristics [33,34,35], and good formability [36], along with compatibility with advanced coating technologies [37,38] and material functionalization methods [39,40,41]. Such comprehensive processability often surpasses that of nickel- or cobalt-based superalloys, particularly in machining operations and joining processes [42,43,44,45], making them more practical for industrial-scale manufacturing.

Research interest in Fe-based alloys, as evidenced by the publication trends shown in Figure 1c,d, demonstrates consistent growth, with a clear evolution from traditional research themes like steel metallurgy, mechanical properties, and microstructural analysis toward emerging frontiers such as shape memory alloys, powder metallurgy techniques, amorphous material development, activity coefficient studies, and hyperfine interaction investigations. The properties of Fe-based HTAs can be precisely engineered through strategic alloying with elements such as Cr, Ni, Mo, Al, and Si [23,46,47], enabling tailored improvements in oxidation resistance and mechanical stability under extreme operating conditions. This adaptability renders them suitable for demanding applications including gas turbine components [19,48], HT exhaust systems, steam power generation infrastructure [46], and nuclear reactor pressure vessels [35].

The selection of Fe-based alloys for this review is justified by their exceptional cost–performance ratio, versatile processability, and rapid technological advancements. Moreover, emerging strategies such as high-entropy alloy design, additive manufacturing techniques, and machine learning-assisted development are transforming the field of iron-based superalloys. This review synthesizes advances in iron-based high-temperature alloys while addressing key challenges and opportunities to guide future breakthroughs in extreme environment performance.

## 2. Alloying Strategies

Fe-based alloys are classified based on their primary alloying element (Me), structure, and intended application. This section focusses on the influence of alloying elements. The performance of Fe-based HTAs is heavily influenced by the choice of alloying elements. The key elements commonly used in Fe-based alloys are chromium (Cr) [49,50,51], aluminum (Al) [52,53,54,55], silicon (Si) [56,57,58], nickel (Ni) [59], and zirconium (Zr) [60]. Besides these, other elements such as molybdenum (Mo) [61], vanadium (V), cobalt (Co), titanium (Ti), Tungsten (W), and manganese (Mn) [62,63] are also used to optimize the high-temperature properties of Fe-based alloys. Nitrogen (N) also plays a crucial role in the nitriding of Fe-based alloys, where alloying elements like Ti, V, Al, Si, Cr, and Mo form nitrides (MeNx) in the matrix. The resulting properties of the nitrided alloy depend on the alloy composition, the crystal structure [64] and morphology of the nitrides, and the nitriding depth, as summarized by Steiner and Mittemeijer [65].

**Figure 2:**
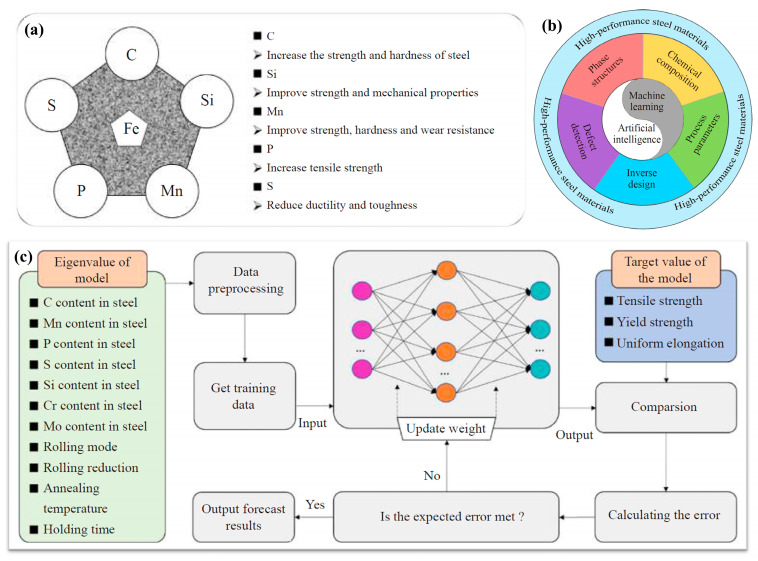
(**a**) Typical five alloying elements used in Fe-based alloys and their effects on mechanical performance, Reprinted from Ref. [66], Open access. (**b**) Application of AI and ML techniques in steels, reprinted from Ref. [72], Copyright © 2023, Springer Nature. (**c**) Schematic diagram of a prediction model for Fe–C alloys based on the backpropagation neural network model, Reprinted from Ref. [66], Open access.

Du et al. [66] provide a concise overview (Figure 2a) of how fundamental alloying elements—C, P, S, Si, and Mn—affect the microstructure and mechanical properties of iron-based alloys. Among them, C plays a crucial role in both ironmaking and steelmaking processes due to its essential function as a reductant in the extraction of iron from its ores. Moreover, carbon significantly influences the microstructure, mechanical properties, and HT behavior of Fe-based alloys, making it a key factor in determining their performance [67,68]. Fe-based HTAs can be classified based on their carbon content into three main categories: low-carbon alloys, medium-carbon alloys, and high-carbon alloys. Low-carbon alloys excel in oxidation resistance and thermal stability, making them ideal for components exposed to high-temperature gases. Wear resistance increases with carbon content for Fe-based HTAs (such as martensitic secondary hardening steels) [69]. Artificial intelligence and machine learning (AL/ML)-driven alloy research is gaining significant attention [2]. By leveraging data-driven modeling, AI/ML accelerates the prediction of Fe-based alloy properties and optimizes compositions [70,71,72,73]. In the context of iron–carbon alloying design strategies (Figure 2b), the application and development of machine learning methods in phase structure identification, process parameter optimization [74], and mechanical properties optimization based on composition and microstructure, as well as the composition inverse design technology and surface damage detection method of iron and steel materials, have been thoroughly introduced and discussed [72]. Further, fundamental alloying elements and other features are used as input parameters and then trained through a backpropagation neural network model, as shown in Figure 2c [66,72].

The introduction of Cr and Al can enhance the oxidation resistance by forming a protective oxide layer on the surface, which is crucial for high-temperature applications like gas turbines and exhaust systems [49,52]. The addition of Si improves the overall resistance to oxidation, and increases the strength of the alloy at high temperatures [75]. Cast irons with a high silicon level are widely used when equipment is being handled in sulfuric acid environments. Though primarily a component in nickel-based alloys, small amounts of Ni are sometimes added to Fe-based alloys to improve thermal stability and creep resistance. Further, the refractory metals (Mo, W, and V) enhance the high-temperature strength, creep resistance, and overall thermal stability of the alloys [75,76], which is also critical for maintaining structural integrity under prolonged high-temperature stress. Moreover, Yang et al. [35] found that the solute elements (Mn, Ni, Si) played an important role in the grain structure, hardness, irradiation hardening, and defect clustering behavior of Fe-based model alloys.

The incorporation of rare earth metals has been demonstrated to significantly enhance the performance characteristics of high-temperature alloys [54,77,78,79,80]. A notable example is shown in the work of Qi et al. [77], who established that yttrium (Y) additions markedly improve high-temperature steam oxidation resistance in FeCrAl alloys. This enhancement primarily occurs through the stabilization of the protective surface oxide film. The same research group further investigated this phenomenon through first-principles calculations [81], revealing the atomic-scale mechanisms by which Y improves oxidation resistance. Their theoretical analysis complemented the experimental findings, providing fundamental insights into the role of Y in surface oxide stabilization.

As for the alloying of Fe-based alloys, significant attention has been paid to the synergistic effects of various alloying elements [82,83]. These synergistic interactions, achieved through the careful selection and combination of two or more elements, are essential for optimizing the performance of the alloy [84]. Each alloying element contributes distinct properties, but their combined effects often result in enhanced mechanical, thermal, and chemical characteristics that surpass the individual contributions of each element. For instance, Cr is frequently used in Fe-based alloys to enhance oxidation and corrosion resistance by forming a stable, protective Cr_2_O_3_ layer on the surface. When combined with Al [85], Mn [86], and Si [47], the protective effect is amplified. The synergistic interaction of Cr and Al extends the durability of the alloys in harsh environments [87,88], known as Fe–Cr–Al alloys [61,89,90,91], making them suitable for applications such as catalyst support [24], as well as cladding in nuclear reactors [23,92], gas turbines [93], and heat exchangers [94].

The synergistic combination of Ni and Mo significantly enhances creep resistance and solid solution strengthening in Fe-based alloys. Ni improves thermal stability and fracture toughness [59], while Mo enhances the resistance to deformation under prolonged high-temperature stress [76]. When alloyed with Cr and/or Ni, Co further contributes to improved HT strength and oxidation resistance [95]. Studies have demonstrated that Cu [63,96], Al [63,97], Mn [63], Ti [22,98] and Mo [95,99] effectively promote precipitate formation, further optimizing alloy performance. These precipitates serve as potent obstacles to dislocation motion, significantly enhancing strength and creep resistance. For instance, Ti and Mo enhance high-temperature strength and creep resistance by refining grain structures and stabilizing precipitates [100]. Another notable example is the strategic incorporation of Ti and V with other alloying elements [101]. Ti facilitates grain refinement and stabilizes carbide/nitride formation, while vanadium strengthens the alloy via fine vanadium carbide precipitates [76,101]. These elements collectively stabilize the microstructure by promoting the formation of beneficial intermetallic phases, including Ni(Al, Fe) [102], NiAl [103,104], Ni_3_Ti [95], Ni_2_TiAl [103,105,106], Ni_3_(AlTi) [107], and CoAl [108], as well as other precipitate phases [22,109,110,111]. Together, these elements substantially improve both high-temperature strength and wear resistance in Fe-based alloys. It is important to note that the NiAl phase contributes to brittleness and promotes intergranular fracture in Fe–Ni–Co–Al-based alloys [22]. To address the dual challenges of the poor room-temperature ductility and inadequate creep resistance above 600 °C shown by NiAl-strengthened ferritic steels, a thermodynamic calculation-guided alloy design approach was employed [112]. Through this method, an optimized Fe-10Cr-2Mo-11Al-13.5Ni (at. %) composition was developed and experimentally evaluated. The results demonstrate that this alloy achieves an effective balance between room-temperature ductility and HT creep performance, successfully mitigating the inherent limitations of NiAl-strengthened systems.

**Figure 3:**
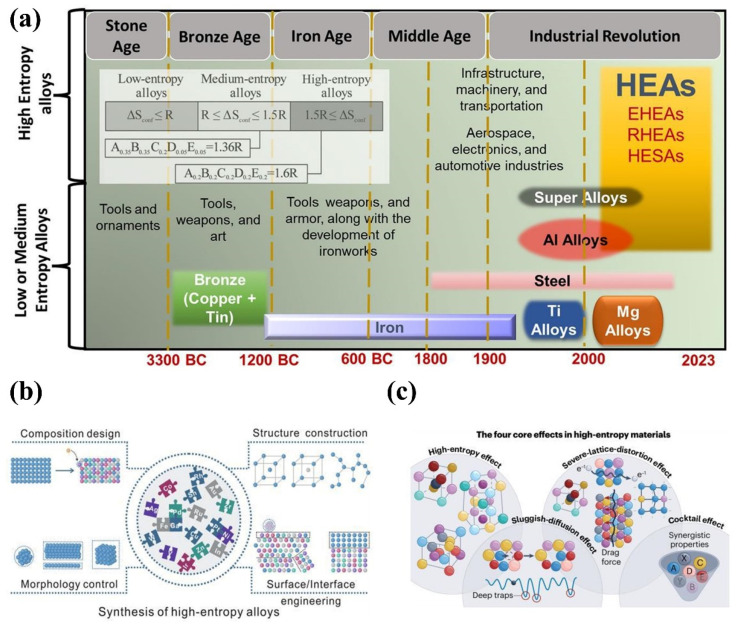
(**a**) Schematic diagram of the various developmental stages of metal-based materials according to configurational entropy. Reprinted with permission from Ref. [113]. Copyright © 2025, Springer Nature. Schematic diagram of the controllable synthesis strategy (**b**) Reprinted with permission from Ref. [115] Copyright © 2024, The Royal Society of Chemistry. Four core effects of HEAs (**c**) Reprinted with permission from Ref. [116] Copyright © 2024, Springer Nature. (EHEAs = eutectic high-entropy alloys, RHEAs = refractory high-entropy alloys, HESAs = high-entropy superalloys).

The development of metal materials has been marked by a significant increase in chemical complexity, driven by the need for advanced properties in demanding applications (Figure 3a) [113,114]. This diversification has led to the creation of novel materials, including medium-entropy alloys (MEAs), high-entropy alloys (HEAs), and other chemically complex systems. The strategy design of synthesizing high entropy mainly includes the regulation of the composition, morphology, structure, and surface/interface engineering (Figure 3b) [115]. The superior performance of these materials can be explained by four key effects: the high entropy effect, the sluggish diffusion effect, the lattice distortion effect, and the cocktail effect (Figure 3c) [116].

The synergistic effect also plays a critical role in high-entropy alloying approaches, where multiple principal elements are combined in near-equimolar proportions [114,117,118,119,120,121]. Among these, Al, Co, Cr, Cu, Fe, Mn, Ni, and Ti elements are the most commonly used for high-entropy alloys (HEAs) [122]. For example, the combination of Fe, Ni, Co, Cr, and Al in high-entropy configurations enhances oxidation resistance [123]. Research has demonstrated that the AlCoCrFeNi HEA containing refractory elements exhibits superior high-temperature oxidation resistance compared to traditional refractory alloys (825 Ni-based alloy and 2205 duplex stainless steel) with a similar Cr content at 1000 °C. These synergistic interactions produce a balanced combination of properties, making such alloys particularly suitable for high-temperature structural applications [124,125].

Compared to conventional high-temperature (HT) alloys, the HT properties of HEA consisting of the refractory element can be further enhanced through several mechanisms: increased anti-phase boundary energy from high alloying concentrations, restrained grain boundary sliding due to sluggish diffusion effects, and reduced recrystallization driving forces resulting from severe lattice distortion [118,120]. Furthermore, incorporating light alloy elements benefits both lightweight design [126] and plasticity optimization [120]. Beyond high-temperature performance, the multi-principal-element design also significantly improves cryogenic temperature performance [127,128,129].

Recently, iron-based high-entropy alloys (HEAs) have attracted significant attention due to their cost-effectiveness [127,130,131]. For example, Fe_50_Mn_20_Al_20_Cr_5_Ti_5_ (Alloy 1) and Fe_50_Mn_25_Al_15_Cr_5_Ti_5_ (Alloy 2) were identified through high-throughput CALPHAD computational screening [132]. Both alloys demonstrate excellent tensile and compressive properties at elevated temperatures, with yield strengths of 607 MPa and 643 MPa, respectively, at 700 °C. These properties result from precipitate strengthening by nano-scale L2_1_ phases and stabilized fine microstructures. Similarly, a BCC-based Fe_72.4_Co_13.9_Cr_10.4_Mn_2.7_B_0.34_ HEA steel achieves an exceptional balance of strength and ductility [133]. The enhanced strength originates from three synergistic mechanisms: optimized compositional modulation, refined BCC-structured grains, and the grain boundary segregation of interstitial boron. The remarkable plasticity stems from multiplanar dislocation slips, coherent interfaces, and shear band formation. This multi-element alloying strategy not only simultaneously improves strength, ductility, and oxidation resistance, but also provides a versatile platform for developing advanced materials to meet increasingly demanding high-temperature applications [134].

In summary, optimizing Fe-based HT alloys depends critically on harnessing synergistic effects among alloying elements. Through the strategic selection and combination of elements such as Cr, Ni, Al, Mo, and Ti, these alloys achieve enhanced mechanical strength, oxidation resistance, and thermal stability. These synergistic improvements are essential for developing materials capable of withstanding extreme operational conditions in demanding high-temperature applications across aerospace, energy, and manufacturing sectors.

## 3. Fabrication and Processing

The manufacturing methods for Fe-based HTAs include melting and casting [135], powder metallurgy (PM) [109,136], vacuum induction melting (VIM) [77,137,138,139], arc and electroslag remelting, spray deposition, laser cladding, arc welding and electro-slag welding (ESW), diffusion bonding, chemical vapor deposition (CVD), physical vapor deposition (PVD), friction stir processing [140], and additive manufacturing (AM) [141]. The choice of alloying method depends on several factors, including the desired properties of the alloy, the production scale, and the application requirements. For Fe-based HTAs, selecting the right alloying method is crucial for achieving the necessary balance of strength, oxidation resistance, and thermal stability, while also ensuring cost-effectiveness and manufacturability. These methods enable the fine-tuning of alloy properties to meet the demanding conditions of industries such as aerospace, power generation, and automotive.

Each of these methods offers distinct advantages depending on the specific application requirements and desired properties of Fe-based HTAs. Whether employing conventional melting and casting techniques or advanced processing methods such as powder metallurgy (PM) and additive manufacturing (including laser cladding and powder-bed technologies), the alloying process plays a pivotal role in developing Fe-based materials capable of withstanding extreme high-temperature conditions. The selection of processing method is ultimately determined by multiple factors including production scale, compositional complexity, and final performance specifications. While this review primarily focuses on the effects of the alloying strategy due to space constraints, it is important to acknowledge that other critical factors—including manufacturing and design approaches [142,143,144,145], machining processes [18], heat treatment protocols [146,147,148], surface treatments [149], and protective coatings [150,151]—significantly influence the ultimate properties of these materials.

Advanced high-strength steels (AHSS) constitute a specialized category of steel alloys characterized by substantially enhanced mechanical properties, with minimum yield strengths exceeding 460 MPa—markedly superior to conventional carbon steels [152]. The AHSS family comprises several distinct classifications based on microstructure and processing techniques: dual-phase (DP) steel [153], transformation-induced plasticity (TRIP) steel [154,155], press-hardened steel (PHS), twinning-induced plasticity (TWIP) steel [156], and complex-phase (CP) steel, among others [157,158]. Building upon this foundation, ultra-high-strength steels (UHSS) further push performance boundaries through sophisticated alloying strategies, incorporating multiple elements to optimize strength, ductility, and crash resistance [159,160,161]. For instance, with alloying additions of 3 wt. % and 4 wt. % Cu, a typical Fe-22Mn-0.6C TWIP steel demonstrates improved properties through a grain-refinement strategy [96]. The alloying elements are typically treated as the solution soluted in the BCC Fe phase. Lin et al. [162] showed that the introduction of alloying elements (Ni, Mo, Cr, and C) markedly amplifies the elastic modulus of Fe-based alloys by first-principles phonon calculations. And the interstitial solid solution of C provides a more significant enhancement to the elastic modulus, while Ni, Mo, Cr, and C elements notably enhance the anisotropy of the Fe-based alloy [162]. Recently, a machine learning (ML) framework was developed to predict and optimize the stretch-flangeability of AHSSs by establishing composition–microstructure–property correlations, utilizing datasets encompassing 212 steel conditions [163].

**Figure 4:**
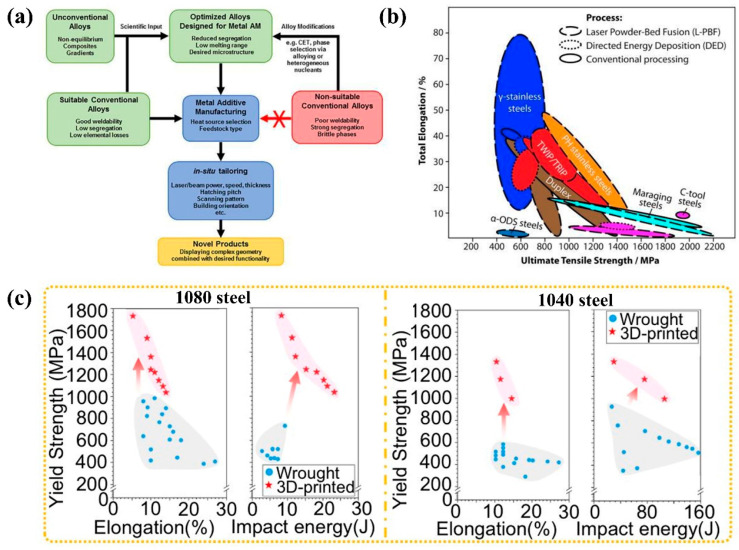
(**a**) A schematic diagram of alloy design for metal additive manufacturing, Reprinted with permission from Ref. [164] Copyright © 2018 John Wiley and Sons. (**b**) A comparison of properties obtained by conventional processing and by the two 3D-printed processes (DED and L-PBF) (the kind of steel is denoted by field color, while the field border indicates the method of production), Reprinted with permission from Ref. [169] Copyright © 2019 Elsevier. (**c**) Mechanical properties of 3D-printed 1080 (left) and 1040 (right) steels in comparison with their conventionally wrought counterparts, Reprinted with permission from Ref. [170] Open access. (CET = Columnar to equiaxed transition, L-PBF = laser powder-bed fusion, ODS = oxide dispersion-strengthened, C-tool steels = carbon-bearing tool steels, PH = precipitation hardening).

When iron-based superalloys meet additive manufacturing or 3D printing technology, it gives brings about new opportunities [92,159]. Fe-based alloys, together with Ti-based alloys, Al-based alloys, and Ni-based alloys, are the most common alloys produced via AM technology [164]. The use of 3D printing technology for Fe-based HTAs is an area of growing interest [164,165,166,167,168]. Figure 4a displays a schematic diagram of alloy design for 3D-printed metal. Optimizing alloys for metal AM involves balancing multiple factors to achieve desired mechanical properties, printability, and microstructure. The ideal AM alloy minimizes segregation and cracking (e.g., short freezing ranges), leverages rapid solidification for fine microstructures, and aligns strengthening mechanisms (precipitation, solid solution, etc.) with the characteristics of a base metal. This method allows for the production of complex geometries and custom components for HT applications [164,169].

Figure 4b presents a schematic overview of basic mechanical properties of widely used steels processed through both AM and conventional methods [169]. A comparative study of 15-5 PH stainless steel produced by additive manufacturing (direct metal laser sintering) versus traditional manufacturing (TM, wrought) revealed that AM-processed alloys exhibit superior Vickers microhardness [171]. Research on Fe-based alloys has primarily focused on steel materials [159,164], with particular emphasis on austenitic stainless steel 316L [30,142,166,172,173,174,175,176,177]. Previous studies [164,175] demonstrate that AM-produced 316L achieves a higher yield and ultimate tensile strength compared to its conventionally wrought and annealed counterparts. The enhanced yield strength and hardness are attributed to the intragranular cellular segregation network structure and randomly distributed oxide nano-inclusions formed during selective laser melting (SLM), along with the refined dendrites resulting from rapid solidification under direct metal deposition (DMD). The observed reduction in ductility can be explained by the presence of “track–track” and “layer–layer” melt pool boundaries [164].

A comparative analysis of yield strength–elongation and yield strength–impact energy relationships between 3D-printed plain carbon steels (1080 and 1040 steels) and conventional wrought steels (Figure 4c) has shown improved strength–ductility–toughness performance in the AM specimens. Notably, the 3D-printed 1080 steel achieved a yield strength of 1773 MPa, while the 1040 grade reached 1340 MPa [170]. The laser wire direct energy deposition technology has found widespread application in complex part manufacturing and welding repair applications. This technique has been successfully employed to fabricate lean duplex stainless steel 2101 using underwater laser wire direct energy deposition [178].

AM technology has received extensive attention for use in materials functionalization. For example, complex-shaped Nd_2_Fe_14_B magnets [179] and Fe-based soft magnetic materials [180] have been successfully fabricated using the SLM process. The AM-processed Fe-based soft magnetic materials exhibit superior magnetic properties compared to those of the samples produced by traditional molding techniques [180]. Functional gradient Fe-based alloys can be readily manufactured via the AM process [142], as the process follows a layer-by-layer stacking principle [181]. The AM technology enables the precise fabrication of porous or lightweight Fe-based alloy structures, further optimizing their functional applications in biological materials [182,183], energy-absorbing materials [181,184], catalytic supports [24,185], shape memory alloys [184], and other specialized applications [167,181].

The steel industry is a major contributor to global carbon emissions, driving the need for sustainable and eco-friendly fabrication and processing methods for Fe-based alloys [186]. Sustainability-related research topics in iron and steel production have been thoroughly summarized by Raabe [186]. Recent advances focus on reducing energy consumption, minimizing waste, and utilizing renewable resources while maintaining material performance. The transition toward the sustainable manufacturing of Fe-based alloys demands innovative approaches to reduce environmental impacts while maintaining material performance. A promising direction is hydrogen-based direct reduction (H-DRI) [187,188], which replaces carbon-intensive coke with green hydrogen as the reducing agent for iron ore. A sustainable method, turning oxides directly into green alloys in bulk forms through a one-step solid-state process, was reported by Wei et al. [189]. Further, sustainable bulk nano-structured porous alloys were produced via the reactive vapor-phase dealloying–alloying synthesis route by same group [190]. Complementing this shift in primary production, electric arc furnaces (EAFs) powered by renewable energy are enabling the large-scale recycling of steel scrap with significantly lower carbon footprints [186,191,192]. When combined with alloy designs that avoid hazardous or difficult-to-recycle elements, this approach supports a circular economy model for Fe-based materials [193]. The processing stage also offers sustainability gains through advanced techniques like additive manufacturing [194], which minimizes material waste through near-net-shape fabrication, and solid-state methods such as friction stir welding [140] that eliminate energy-intensive melting steps. Atypical ferroalloys, e.g., rare earth element permanent magnets (NdFeB), are widely used in a multitude of industries, especially in electric motors for electric and hybrid vehicles and wind turbines [195]. The recycling potential of NdFeB permanent magnets and the identified physical and metallurgical separation and recovery technologies have been attracting more and more attention [196,197].

Further improvements have emerged from strategic alloy development, whereby critical raw materials like cobalt and rare earth elements are substituted with more abundant alternatives. For instance, new generations of maraging steels have achieved high strength without relying on Co or Mo [198]. A sustainable ultra-high-strength maraging steel (Fe_18_Mn_3_Ti), with the abundant Mn as a major alloying element, was obtained through controlled solute segregation and α-Mn nanoprecipitation [198]. Concurrently, digital tools like machine learning optimize energy use in rolling and heat treatment processes [71,199], while lifecycle assessments guide material selection based on comprehensive environmental metrics.

**Figure 5:**
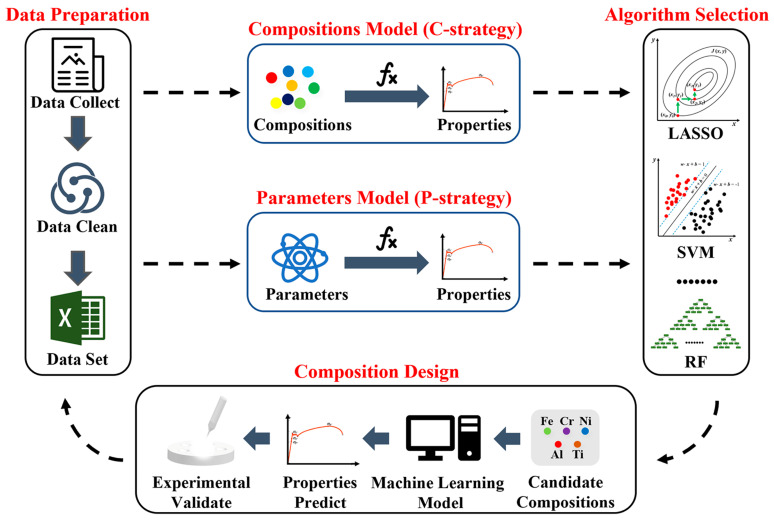
Schematic diagram of composition design via machine learning for Fe–Cr–Ni–Al/Ti multi-principal element alloys, Reprinted with permission from Ref. [97] Copyright © 2019 Elsevier.

Recently, artificial intelligence and machine learning (AI/ML) have been used in material discovery and design [200,201,202,203]. AI/ML are transforming iron-based alloy design by enabling the rapid exploration of vast compositional and processing spaces, reducing trial and error, and uncovering novel materials [2,44,68,177,204,205,206,207,208]. An ML framework for designing high-performance multi-principal element alloys (Fe-Cr-Ni-Al/Ti) is shown in Figure 5 [97]. The process follows four key steps: data preparation (collecting composition–property datasets), feature selection (choosing input variables), model selection (picking an ML algorithm), and model application (using the model to predict optimal compositions). Two distinct training strategies are highlighted: the C-strategy, which directly uses alloy compositions as model inputs, and the P-strategy, which relies on composition-derived features (e.g., thermodynamic or structural descriptors). The study demonstrates how this ML-driven approach efficiently identifies promising alloys, focusing on optimizing strength and toughness, and bypassing traditional trial-and-error methods [97]. As datasets grow and algorithms improve, AI/ML will play an even greater role in optimizing process conditions [71], predicting mechanical properties [209,210,211], and designing the next generation of advanced steels [2], high-entropy alloys [8,212], functional iron-based materials [213], and others [72].

## 4. High-Temperature Properties

The high-temperature (HT) properties of Fe-based alloys are critical for applications in aerospace, power generation, chemical processing, and other industries where materials are exposed to extreme conditions. Research on Fe-based alloys and steels has predominantly focused on chemical corrosion [214,215,216], biological corrosion [217,218], and performance in complex environments [214,219,220]. When subjected to elevated temperatures, Fe-based alloys exhibit a range of essential performance characteristics, including oxidation and corrosion resistance, mechanical strength, creep resistance, thermal stability, and microstructural stability. These properties collectively determine their suitability to demanding high-temperature applications.

### 4.1. Oxidation and Corrosion Resistance

Fe-based alloys, particularly austenitic stainless steels (types 304, 316, and 347), have served as nuclear fuel cladding materials since 1951 [85]. The alloying strategy significantly influences the oxidation and corrosion resistance of these metals. Protective oxide layers such as Cr_2_O_3_ [221], Al_2_O_3_ [222,223] and FeCr_2_O_4_ [52] form on the alloy surface, effectively preventing degradation in both oxidizing and carburizing environments. Previous investigations [51,221,224,225] have demonstrated that adding Cr to form an Fe–Cr alloy enhances oxidation resistance at elevated temperatures. Additional elements including Al [54,222,226], Ni [59,84], Co [227], Zr [60], Mn [228], and others [229] have been employed to further improve HT oxidation and corrosion resistance performance. Building upon the Fe–Cr alloy foundation, subsequent developments incorporated new alloying elements such as Ni [224], Al [50], Y [50], Ti [47,146], Si [47], and Mo [230]. In recent years, FeCrAl alloys have emerged as particularly outstanding due to their exceptional HT resistance properties.

**Figure 6:**
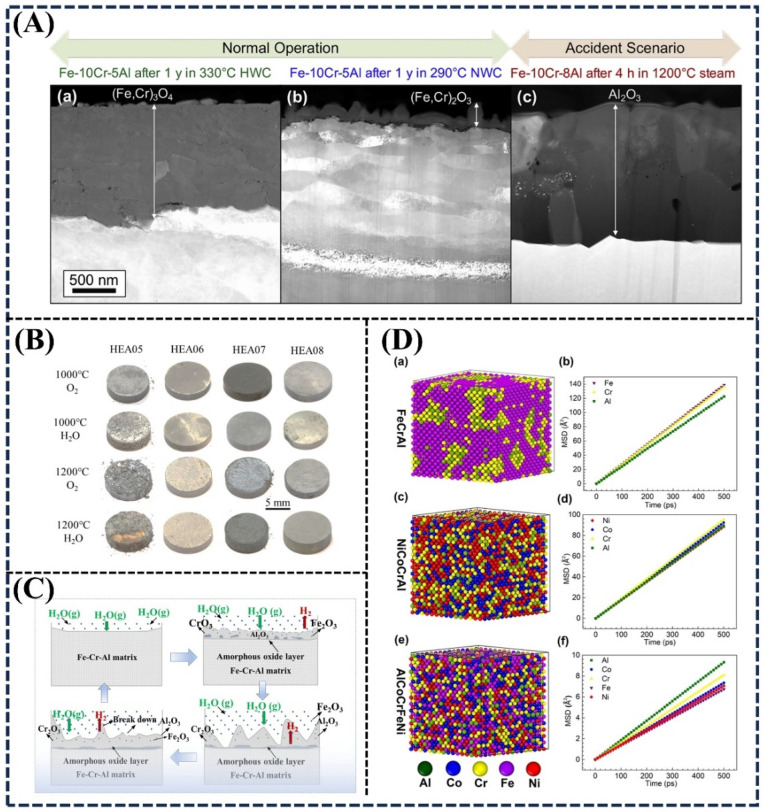
(**A**) Various protective films ((**a**,**b**) Cr-rich spinel or hematite films, (**c**) Al_2_O_3_ film) formed on the surfaces of FeCrAl alloys in various environments: (**a**,**b**) in low- and high-oxygen-activity aqueous environments, (**c**) under HT steam oxidation (1200 °C, 4 h), Reprinted with permission from Ref. [85] Copyright © 2018 Elsevier. (**B**) Surface morphology of the HEA alloys after the isothermal oxidation test for 20 h, Reprinted with permission from Ref. [257] Copyright © 2021 Elsevier. (**C**) Schematic illustration of the surface steam oxidation mechanism in an Fe–Cr–Al alloy, Reprinted with permission from Ref. [87]. Copyright © 2022 Elsevier (**D**) Hybrid molecular dynamics and Monte Carlo simulations of atomic migration behavior in the Al-depletion layer for FeCrAl alloy (**a**,**b**), NiCoCrAl alloy (**c**,**d**), and AlCoCrFeNi HEA (**e**,**f**) at 1100 °C, Reprinted with permission from Ref. [244] Copyright © 2022 Elsevier.

The development status for three typical accident-tolerant fuel cladding materials (coated Zr-based cladding, FeCrAl cladding, and SiC/SiC cladding) was thoroughly summarized in a previous review [85]. A comparative analysis of FeCrAl alloy corrosion behavior in a light water reactor (LWR) coolant environments is presented in Figure 6A(a,c). During normal operation and anticipated operational occurrences (including both hydrogen water chemistry (HWC) and normal water chemistry (NWC)), protective Cr-rich oxide films dominate after one year of exposure. In contrast, accident scenarios involving HT steam oxidation (Figure 6A(c)) primarily yield alumina-based protective layers. Figure 6B exhibits optical micrographs of oxidized HEAs with various compositions (HEA06 = Al_0.5_CrFeNiMn, HEA06 = Al_0.5_CrFeNiCo, HEA08 = Al_0.5_CrFeNiCo + Zr, HEA08 = Al_0.5_CrFeNiCo + Y). The HEA08 samples consistently formed compact, smooth oxide layers without macroscopic exfoliation, while other HEA alloys exhibited varying degrees of oxide particle spallation after HT oxidation. These results unequivocally demonstrate the critical role of alloy design in determining HT isothermal oxidation performance [85].

Under irradiation, Fe-based nuclear materials undergo progressive degradation as atomic displacements generate defects that evolve into clusters and voids, while transmutation-produced helium and hydrogen exacerbate damage [231]. These effects cause radiation hardening, swelling, and segregation—all thermally accelerated—ultimately compromising mechanical performance [232,233,234,235,236]. Current research focuses on radiation-tolerant designs including nanostructured alloys [232,237], ODS steels [238,239], and HEAs [235,236,240,241,242] that engineer microstructures to control defect accumulation. Cheng et al. [240] and Tan et al. [241] systematically evaluated the irradiation responses of HEAs, highlighting their unique potential utility in nuclear environments. The chemical complexity and lattice distortion inherent to HEAs promote enhanced defect recombination, suppressing void swelling and radiation-induced hardening compared to conventional alloys. While their unique properties offer potential radiation resistance, significant gaps remain between laboratory results and practical implementation. The field needs rigorous studies that simultaneously address fundamental radiation behavior, engineering challenges, and economic feasibility to determine if HEAs can evolve from scientific novelties to viable nuclear materials [242].

Figure 6C gives the mechanism diagram of the surface steam oxidation of FeCrAl alloys [87]. The study has revealed that Al_2_O_3_ formation plays the dominant role during HT steam oxidation between 1000 °C and 1300 °C. Cr also has a positive impact on surface-oxidizing film formation [49], particularly by enhancing long-term oxidation resistance through reduced oxidation weight gain rates. FeCrAl alloys have garnered considerable attention for use in nuclear fuel cladding applications due to their superior HT oxidation resistance compared to conventional zircaloy [23,27,92]. Further comparative studies under actual operating conditions (500–520 °C for 6–12 months in a loop seal region of an 85 MW_th_ waste-fired CFB boiler in Sweden) showed that FeCrAl alloys outperform both austenitic stainless steels and Ni-based alloys in terms of oxidation resistance [94]. Beyond experimental investigations, HT corrosion behaviors have been successfully simulated using the Calphad (Calculation of Phase Diagrams) approach [243].

Hybrid molecular dynamics (MD) and Monte Carlo (MC) simulation serve as an effective tool for investigating elemental migration dynamics behavior. To compare diffusion coefficients among three alloys (FeCrAl, NiCoCrAl, and AlCoCrFeNi HEA) at 1100 °C [244], hybrid MD/MC simulations were conducted using the large-scale atomic/molecular massively parallel simulator package. Figure 6D demonstrates the simulation results of the Al-depletion layer for FeCrAl alloy (Figure 6D(a,b)), NiCoCrAl alloy (Figure 6D(c,d)), and AlCoCrFeNi HEA (Figure 6D(e,f)) at 1100 °C. The HEA system, exhibiting the highest configurational entropy, demonstrates maximum atomic chaos, followed by NiCoCrAl and the FeCrAl alloy system (Figure 6D(a,c,e)). The increasing rate of mean square displacement (MSD) of the different elements reflects their diffusion rates (Figure 6D(b,d,f)). The Al diffusion coefficients follow the order FeCrAl > NiCoCrAl > HEA system. The significantly lower diffusion coefficient (approximately one order of magnitude) in AlCoCrFeNi HEA indicates that inward oxygen diffusion dominates Al_2_O_3_ scale growth. This inward oxygen diffusion promotes the formation of columnar Al_2_O_3_ grain structures, while reduced outward Al diffusion decreases oxidation rates and inhibits interface rumpling, thereby enhancing oxidation resistance [244].

To further improve HT performance, minor elements including Mo [61], Y [77], Nb [245], Ta [246], Si [84], and so on [23,91,238] have been introduced into the FeCrAl alloy. Recent advances have employed multi-component [247] and high-entropy designs [248,249], along with strategies like oxide dispersion strengthening (ODS) [238,250], machine learning [2,251], and optimized machining processes [251,252]. Beyond HT steam environments, the influences of alloying elements on FeCrAl(Si)/(FeCrNi) alloys in corrosive environments containing KCl (or K_2_CO_3_) + HCl (600 °C, 168 h) were also investigated by atom probe tomography [84]. This further confirmed that alloying elements benefit corrosion protection through the formation of inward-growing heterogeneous scales.

The corrosion behavior of Fe-based alloys in various molten salt environments has been extensively investigated, with studies on Li_2_CO_3_-Na_2_CO_3_-K_2_CO_3_ with TP347H stainless steel at 650 °C [253], solar salt (60%NaNO_3_-40%KNO_3_) with ferritic steels at 400–600 °C [254], chloride salt (NaCl–KCl) with 316L stainless steel at 800 °C [255], and Li_2_CO_3_–Na_2_CO_3_–K_2_CO_3_ and LiCl–Li_2_O with Fe–36Ni alloy at 650 °C [256]. A comprehensive study of molten carbonate corrosion behavior in TP347H stainless steels with varying Al contents (0–2.5 wt. %) revealed significant performance differences [253]. The steel without Al content (0 wt. % Al) exhibited severe corrosion, demonstrating a weight gain of approximately 9.44 mg/cm^2^ after 1000 h exposure, corresponding to a corrosion rate of 103.07 µm/year. In contrast, the 2.5 wt. % Al alloy showed optimal performance with the lowest weight gain (~6.0 mg/cm^2^) and corrosion rate (75.09 µm/year).

**Figure 7:**
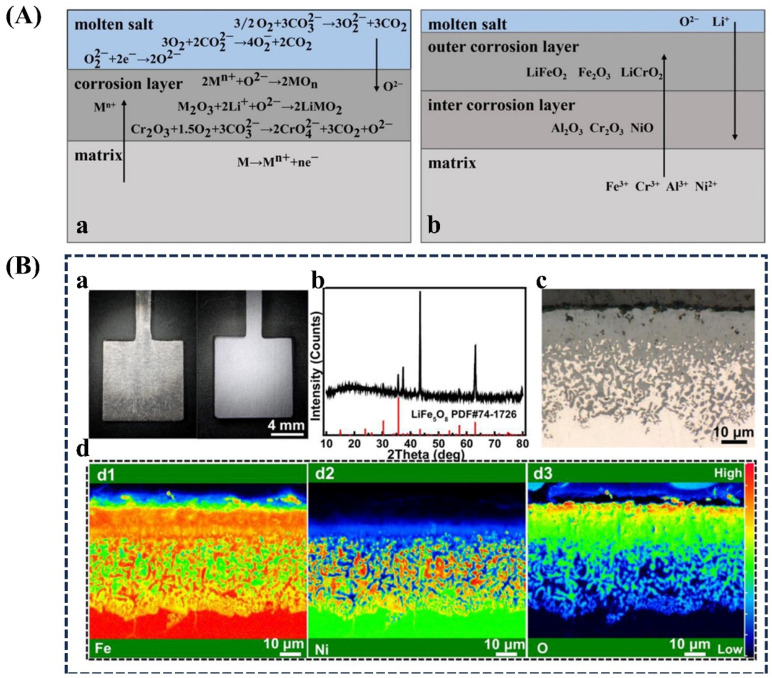
(**A**) Sketch of formation process for corrosion layer: (**a**) the initial stage of corrosion and (**b**) stable corrosion stage, Reprinted with permission from Ref. [253] Open access. (M = Fe, Cr, Ni, and Al). (**B**) Fe-base oxygen evolution electrode in molten LiCl–Li_2_O at 650 °C—(**a**) optical photos of the Fe-36Ni anode before (left) and after pre-oxidation (right), (**b**) XRD data of the oxide scale, optical micrograph (**c**) and EPMA mapping (**d**) of the cross-section of the oxide scale ((**d1**) Fe, (**d2**) Ni, and (**d3**) O) , Reprinted with permission from Ref. [256] Open access.

Figure 7A(a,b) [253] shows a mechanistic diagram of molten carbonate corrosion for Al-modified TP347H steel. In the initial corrosion stage (Figure 7A(a)), the alloy surface undergoes simultaneous reactions with molten carbonate and atmospheric oxygen, forming iron, chromium, and aluminum oxides. These oxides subsequently react with the carbonate melt to generate lithium ferrite (LiFeO_2_) and lithium chromite (LiCrO_2_), along with various spinel phases. Al plays a pivotal role by facilitating the development of a continuous, compact LiFeO_2_ layer that shields the substrate from direct exposure to the corrosive medium. As corrosion processes to the stable stage (Figure 7A(b)), ongoing oxygen diffusion promotes the formation of a protective inner oxide layer primarily composed of Cr_2_O_3_ and Al_2_O_3_, with NiO contributing to the protective barrier. This composite inner layer demonstrates exceptional stability in the molten carbonate environment, significantly enhancing long-term corrosion resistance. The Al–Cr synergy is particularly significant, as Al not only accelerates initial LiFeO_2_ formation, but also improves the density and adhesion of the subsequent Cr_2_O_3_-rich layer. The proposed mechanism (Figure 7A(a,b)) demonstrates that a higher Al content accelerates the development of the protective LiFeO_2_–LiCrO_2_ outer layer, while the Cr-dependent inner oxide layer provides durable protection. This dual-layer system—comprising an outer lithium-containing oxide layer and an inner chromia–alumina-rich barrier—collectively delivers superior corrosion performance in high-temperature molten carbonate environments. These findings highlight the critical importance of optimizing Al concentration to achieve both rapid initial protection and sustained corrosion resistance [253].

### 4.2. Mechanical Properties

The HT mechanical properties of Fe-based alloys (such as carbon steels, stainless steels [258], and other iron-based superalloys [259]) are crucial for elevated-temperature applications. These properties are fundamentally governed by composition, microstructure, and processing [17]. For 15Cr–22Ni–1Nb austenitic heat-resistant steels, the HT ultimate tensile strength (UTS = 346–361 MPa at 650 °C) is 30–40% lower than at room temperature, though appropriate heat treatment can significantly improve elongation after fracture [260]. In FeCrNiAl alloys, the addition of vanadium (0–12 wt. %) introduces coherent hierarchical precipitates (nano-scale Fe_23_Zr_6_ phases within B2–NiAl precipitates), enhancing HT yield strength from 210 MPa (0 V) to 401 MPa (12 V). The HT compressive strain of these ferritic alloys also increases with V content, attributable to the dynamic recrystallization (DRX) behavior [104]. A study of four Fe-based HEAs (Fe_36_Mn_21_Cr_18_Ni_15_Al_10_-Alloy1, Fe_36_Co_21_Cr_18_Ni_15_Al_10_-Alloy 2, Fe_35_Mn_20_Cr_17_Ni_12_Al_12_Ti_4_-Alloy 3, and Fe_35_Co_20_Cr_17_Ni_12_Al_12_Ti_4_-Alloy 4) [261] at room temperature (RT), 400 °C, 600 °C, and 800 °C revealed distinct mechanical responses. The face-centered cubic (FCC) structured Alloy 2 exhibited the lowest yield strength values at both RM (250 MPa) and HT (150 MPa). In contrast, Alloy 4, featuring a body-centered cubic (BCC) matrix with plate-like L2_1_ (ordered FCC) precipitates, demonstrated superior strength characteristics, with 1420 MPa at RM and 795 MPa at 600 °C [261].

HT torsion tests (800–1200 °C) conducted at high strain rates on Fe-based alloys revealed that plastic deformation is governed by a dislocation climb mechanism, providing insights for hot formability studies [262]. Complementary research on Aermet100 steel through isothermal hot compression tests (800–1100 °C) demonstrated that the HT deformation behavior can be accurately predicted using artificial neural network modeling [263]. Zhang et al. [258] investigated the mechanical properties of 316 stainless steels under combined neutron radiation (11.8 dpa) and elevated temperature (320 °C) conditions using in situ X-ray diffraction and ex situ electron microscopy techniques. The study revealed that irradiated specimens exhibited significantly higher yield stresses and reduced total elongations compared to unirradiated controls at both 23 °C and 320 °C.

**Figure 8:**
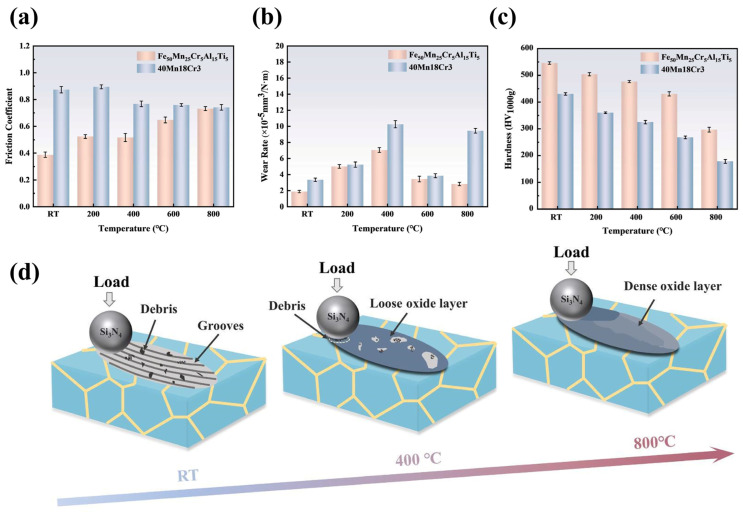
Friction coefficient (**a**), wear rate (**b**), and hardness (**c**) of Fe-based high-entropy alloy and 40Mn18Cr3 steel at different temperatures (RT-800 °C). (**d**) A schematic illustration of the wear mechanisms of Fe_50_Mn_25_Cr_5_Al_15_Ti_5_ alloy at different temperatures, Reprinted with permission from Ref. [264] Copyright © 2025 Elsevier.

Xu et al. [264] systematically investigated the hardness, HT softening resistance, and wear performance (Figure 8a–c) of a multicomponent Fe_50_Mn_25_Al_15_Cr_5_Ti_5_ alloy. The Fe-based alloy demonstrated superior performance compared to Hadfield steel (40Mn18Cr3), exhibiting higher hardness, enhanced resistance to HT softening, and improved wear resistance [264]. At 800 °C, the wear mechanism was attributed to the formation of a dense, protective oxide film on the surface (Figure 8d). Remarkably, Fe-based high-entropy alloys with a composition of Fe-20Mn-15Cr-10V-10Al-2.5C (at.%) achieved Vickers hardness values up to 520 HV [265]. This exceptional hardness originates from localized elemental concentration variations and atomic-scale crystal lattice disorder. The characteristic high-entropy effects—including entropy stabilization, sluggish diffusion, lattice distortion, and cocktail effects—promote the formation of simple solid solution phases instead of complex intermetallic compounds [114]. This optimized multicomponent alloying strategy not only enhances strength, ductility, and oxidation resistance, but also establishes a versatile platform for developing advanced materials to meet growing demands in HT applications.

Four distinct Fe-based alloy systems (austenitic stainless steel, ingot metallurgy M2 tool steel, a complex FeCrCNbMoWB alloy with fine microstructure, and a hypereutectic FeCrCMoNb alloy with coarse microstructure) were investigated for their HT wear resistance using single impact tests [75], continuous impact abrasion tests, and erosion tests [266]. Both RT and HT (600 °C) single impact tests and oblique erosion tests demonstrated strong correlations with material hardness. During continuous impact abrasion testing, the complex FeCrCNbMoWB alloy exhibited superior wear resistance compared to other materials at both RT and 600 °C [75,266]. Recent work by Singla et al. [267] comprehensively reviewed the effects of various alloying elements (B, C, Cr, Mn, Mo, Nb, Si, Ti, V, and rare earth metals) on the wear resistance performance of Fe-based alloys.

The performance of HTAs depends not only on their composition, but also on their long-term degradation resistance. While ferroalloys generally exhibit poor creep resistance above 600 °C, the incorporation of solid-solution strengthening elements such as W, Mo, and Nb enhances their resistance to deformation under prolonged HT stress. Strategic alloying can effectively improve these high-temperature creep properties. For instance, carbon-free FeNiAlCr alloys demonstrate excellent creep-resistant above 600 °C [268]. The addition of aluminum (Al) to TP347H stainless steel substantially improves both strength and elongation at 650 °C compared to the base alloy [253]. Ti additions (0, 2, 4 and 6 wt. %) notably enhance the FeNiAlCrMo alloy, and particularly creep resistance at 700 °C under stresses ranging from 70 to 300 MPa [269]. Subsequent investigations by the same research group examined the effects of minor hafnium (Hf) additions (0.5 wt. %) on FeNiAlCrTi ferritic superalloy [269,270], revealing that creep resistance depends critically on three factors—the volume fraction of precipitates, the lattice misfit between precipitates and matrix, and precipitate coarsening kinetics. Extensive correlation analyses together with ML models were used to predict the creep of HT alloys as well [206]. Oxide dispersion strengthening (ODS) with Y_2_O_3_ dramatically improves the creep resistance of FeCrAl alloys at 1100–1300 °C [271]. The HT creep resistance properties of FeCrAl were significantly improved at 1100–1300 °C. Furthermore, high-throughput design approaches have successfully developed low-activation, high-strength creep-resistant steels for nuclear reactor applications at 650 °C [272].

Additive manufacturing (AM) offers distinct advantages to HT performance compared to conventional manufacturing methods [167]. In the case of 15-5 PH stainless steel [171], AM-produced specimens exhibit superior mechanical properties to their wrought (WST) counterparts, including higher yield strength (790 MPa vs. 610 MPa) and ultimate tensile strength (830 MPa vs. 620 MPa) at 593 °C under a strain rate of 10^−3^s^−1^. Except for the Vickers microhardness [171], the 15-5 PH stainless steel produced by AM showed a higher yield strength (790 MPa) and ultimate tensile strength (830 MPa) than wrought stainless steel (WST, 610 and 620 MPa) at a temperature of 593 °C and a strain rate of 10^−3^s^−1^, despite showing slightly lower ductility. Creep rupture testing at 593 °C under 211 MPa revealed that AM steel outperforms WST material [171], with longer rupture times (157.2 h vs. 121.2 h) and significantly lower minimum creep rates (0.0003%/h vs. 0.038%/h), demonstrating superior creep resistance. Selective laser melting (SLM) has enabled the development of 316L stainless steel with enhanced strength characteristics, achieving a room temperature yield strength of 598.2 MPa and HT yield strength of approximately 350 MPa at 600 °C [273]. These mechanical properties can be further improved through micro- and nano-scale TiC [273] or TiB_2_ additions [274]. Notably, incorporating 10 vol% TiB_2_ nanoparticles produces exceptional strength values, reaching 980.9 MPa at RM and remaining at 833.8 MPa at 600 °C, 702.4 MPa at 700 °C, 401.9 MPa at 800 °C, and 95.8 MPa at 1000 °C [274].

## 5. Applications

Modern high-temperature material systems include superalloys [1,2], refractory metals [275], ceramics [10,276], and composites [277], each offering unique advantages for extreme environments [278,279]. Material selection requires balancing mechanical performance, environmental stability, and cost. While Ni/Co superalloys dominate ultra-high temperature applications, Fe-based alloys remain crucial for moderate temperatures (500–750 °C) due to their cost-effectiveness and manufacturability. Advanced variants like ODS, HEA, and HSS steels are expanding their capabilities. These alloys continue serving critical roles in power generation, petrochemical, and automotive applications, where economics outweigh the need for extreme performance. Ongoing material innovations ensure their continued relevance in high-temperature engineering.

Iron and iron-based alloys have long been recognized as promising anode materials due to their favorable electrochemical properties and cost-effectiveness [280,281,282]. Recent advancements have revealed particularly promising results for iron-based oxygen-evolution electrodes, where an in situ-formed lithium ferrite (LiFe_5_O_8_) surface layer develops during operation [256]. A dense oxide film composed of lithium ferrite (Figure 7B(b)) is formed on the Fe-36Ni electrode after pre-oxidation (100 mA/cm^2^ at 650 °C) (Figure 7B(a–c)). Clearly, a nickel-rich transition layer can be observed between the oxide layer and the bulk metal by electron probe X-ray microanalyzer (EPMA) mapping (Figure 7B(d)), which is attribute to the different diffusion rates of Fe and Ni. And the protective layer plays an important role in HT oxidation resistance and mechanic stability [256]. The HT oxygen evolution reaction performance of the pre-oxidized Fe–36Ni electrode was assessed in molten LiCl–Li_2_O for 20 days, and the calculated consumption rate was only approximately 0.04 cm/year. This dual functionality, which uniquely combines inherent material stability with catalytic enhancement, presents a transformative strategy for developing cost-effective, Earth-abundant electrode materials capable of withstanding prolonged operation in high-temperature electrochemical systems.

Hydrogen embrittlement (HE) is a widely known phenomenon that occurs in various high-strength materials such as high-strength steels, high-Mn steel, Al-based alloys, Ti and Ti alloys, and Mg-based alloys, etc. [283,284,285,286]. HE has detrimental effects in many industrial sectors, such as in municipal wastewater environments [286], the energy sector [287] and hydrogen in transport [288]. The growing demand for metallic materials resistant to both environmental corrosion and HE under HT presents a significant challenge [289]. And both alloy elements and material microstructure modification have a significant impact on the HE of steels [283,290]. It was discovered, for example, that the retarded HE of Fe–Mn–C TWIP (twinning-induced plasticity) steels improves as the Cu element percentage rises [291]. Al addition is an effective solution to improving the HE resistance of high-Mn austenitic steels [292]. Furthermore, multi-principal element design can also improve the hydrogen embrittlement resistance, which has been well summarized by Li et al. [293]. High-strength steels are particularly susceptible to gaseous hydrogen embrittlement (GHE) [294,295]. Neuharth and Cavalli [296] investigated the high-temperature hydrogen embrittlement effect on four types of austenitic stainless steels (310S, 316/316L, and 321). They showed that both the burst strength and the ductility were reduced by exposure to hydrogen at 3.45 MPa and 800 °C for four hours. A small percentage of refractory elements (Mo, Nb, W) can improve the high-temperature (800 °C) resistance to HE in steels and alloys through intermetallic hardening, which retards phase transformations during testing [289].

Amorphous alloys, also known as metallic glasses, are a class of materials that lack long-range atomic order, unlike crystalline materials [297]. Due to their disordered atomic configurations, they exhibit unique physical and chemical properties that differ significantly from those of conventional crystalline alloys [43,297,298]. One of the most attractive features of amorphous alloys is their wide compositional flexibility, which allows for the fine-tuning of their properties [26,299,300]. Alloying elements such as B, P, Si, and C are commonly used to improve the glass-forming ability and tailor the properties of the alloy [26,204]. High-performance Fe-based nanocrystalline alloys have also received widespread attention, especially for use in electrical and electronic devices with good soft-magnetic performance [26,300,301,302]. Good mechanical [303] and high-temperature properties [300,304,305] can be obtained as well.

**Figure 9:**
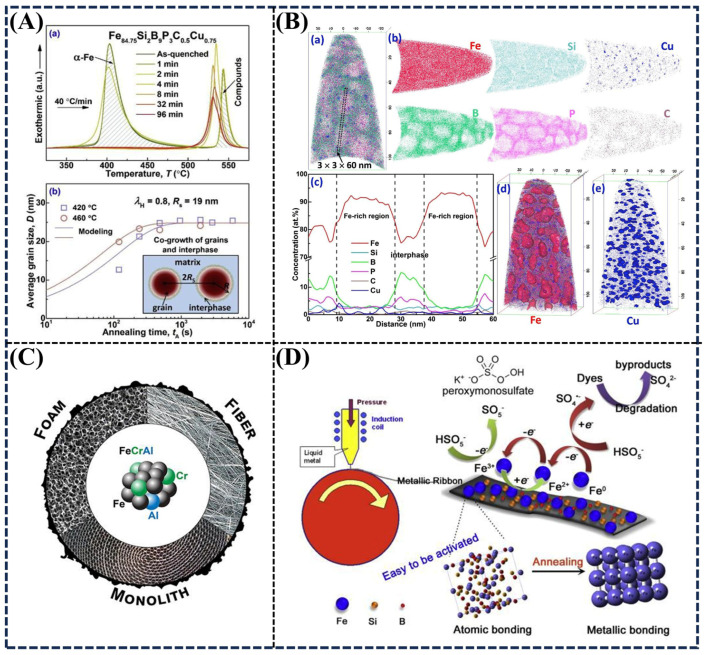
(**A**,**a**) DSC curves for the Fe_84.75_Si_2_B_9_P_3_C_0.5_Cu_0.75_ alloy before (as-quenched) and after annealing at 420 °C for different times (1–96 min). (**A**,**b**) Average grain size (symbols represent the experimental data from XRD and lines are the proposed modeling curves, while the inset is the proposed “dual phase co-growth model”) of the Fe-based nanocrystalline alloys during isothermal annealing at 420 and 460 °C. (**B**,**a**,**b**) APT elemental maps (60 × 60 × 110 nm) of the Fe-based nanocrystalline alloy after annealing at 420 °C for 48 min. (**c**) Concentration depth profile from the selected area (3 × 3 × 60 nm) in (**B**,**a**). (**B**,**d**,**e**) Fe and Cu, respectively, delineated by 90 at.% and 4 at.% iso-concentration surfaces, Reprinted with permission from Ref. [300] Copyright © 2019 Elsevier. (**C**) Three typical structures (monolith, foam, and fiber) of FeCrAl alloys as catalytic supports, Reprinted with permission from Ref. [24] Copyright © 2020 American Chemical Society. (**D**) Schematic illustration of the preparation and proposed PMS activation mechanism of FeSiB amorphous alloys for dye degradation, Reprinted with permission from Ref. [315] Copyright © 2017 Elsevier.

Magnetic alloys have been developed for elevated-temperature applications. Alloying [26,299,306,307,308,309] and heat treatment [300,310,311] play an important role in the development of soft magnetic properties. A previous work [305] indicated that the additions of Co and Ni elements to Nanoperm-type Fe_88_Zr_7_B_4_Cu_1_ soft magnetic alloys dramatically increase the Curie temperature (from 67 to 289 °C) of the intergranular amorphous phase. And low coercivities less than 30 Am^−1^ can be maintained for Fe_77_Co_5.5_N_i5.5_Zr_7_B_4_Cu_1_ nanocrystalline alloys over the temperature range from 50 to 500 °C. The addition of Co and Ni also enhanced the magnetization at different temperatures (25–650 °C) [305]. The high thermal stability of Fe-based nanocrystalline alloys was achieved via the isothermal annealing (420 °C, 0–96 min) of amorphous precursors [300]. When the annealing time is greater than 4 min, the first exothermic peak, ascribed to α-Fe crystallization, disappears (Figure 9A(a)). The disappearance of the first exothermic peak indicates that the precipitation of α-Fe grains levels to a plateau value. A “dual phase co-growth” mechanism has been proposed to understand the kinetics of nano-grain growth (Figure 9A(b)). Further, atom probe tomography (APT) was used to analyze the sample (420 °C, 48 min) so as to predict the grain growth during isothermal annealing. As can be seen in Figure 9B(a,b), typical chemical segregation was observed. The regions with high Fe concentration exhibit a granular shape with an average size of about 20 nm, which is attributed to the α-Fe-like grain, and Si is dissolved in a-Fe like grains. Cu clusters are distributed around the Fe-rich grains with sizes of 2–4 nm, while the metalloid elements (B, P, and C) seemingly play an interphase shielding role in the a-Fe like grains (Figure 9B(c)). The number densities of α-Fe like grains (~2 × 10^23^) and Cu clusters (~1.2 × 10^24^) were respectively estimated from the elemental maps (Figure 9B(d,e)). The formation of nano-sized grains (~20 nm) out of the amorphous matrix gives rise to a synergetic increase in saturation magnetization (B_s_ = 1.84 T), magnetic permeability (μe = ~25,000) and hardness (H = ~15 GPa), with high thermal stability (Figure 9A(a)) [300].

Fe-based alloys can be used as catalytic supports [24,312] and catalysts [313,314]. Typically, amorphous Fe-based alloys or metallic glasses have been used in functional applications as heterogeneous catalysts (e.g., FeSiB for H_2_O_2_ [45], peroxymonosulfate (PMS) [315], and peracetic acid [316] catalytic degradation, FeMoSiB [317] and (Fe_73.5_Si_13.5_B_9_Nb_3_Cu_1_)_91.5_Ni_8.5_ [318] for decolorization). Similarly, the alloying strategy [215,319] and heat treatment [317] have a great influence on the catalytic performance. The stability (chemical stability [298,316], thermal stability) of the alloy is also required in catalyst or catalyst-support applications. A previous review [24] summarized that the FeCrAl alloys can be used as an exceptional catalyst support, especially when used in highly exothermic and endothermic reactions within aggressive chemical environments (>700 °C). The main structures used as catalyst support are in turn monolith, foams, and fibers (Figure 9C), and all are suitable for use in fixed-bed reactors that are easily recycled. The FeCrAl catalyst support can be used in industrial burners, automotive tail gas converters, H_2_ and syngas production, and other applications [24].

Recent work by Magnier and colleagues [313] has demonstrated the development of a highly active and cost-effective oxygen evolution reaction (OER) catalyst for alkaline media. Their study revealed that Fe–Ni alloy substrates spontaneously develop an active surface layer of NiFe oxo-hydroxide species after ageing and activations, which serves as the catalytically active phase. This in situ formation of surface-active sites presents a promising approach for designing efficient OER electrocatalysts, as it combines the stability of alloy substrates with the high-activity characteristic of transition metal oxo-hydroxides. It was found that the NiFe oxo-hydroxide surface layer was determined by the alloying elements and initial atomic Fe/Ni ratio, thus driving the oxygen evolution reaction [313]. The spontaneous surface restructuring phenomenon observed in this system offers new insights into catalyst design strategies that leverage dynamic surface transformations under operational conditions to achieve optimal catalytic performance.

Simultaneously, Fe-based alloys themselves can be used as catalysts. Melt-spun FeSiB glassy ribbons have shown good catalytic reactivity in wastewater remediation [45,315,316]. The amorphous Fe-based alloys were produced by vacuum melt-spinning in a Ti-gettered Ar atmosphere (Figure 9D). the PMS activation mechanism can be explained by the disordered atomic packing structure, which provides catalytically active sites, as well as amorphous zero-valent iron as both the electron donator and the acceptor (Figure 9D) [315]. Further, the inclusion of Si and B could improve the glass-forming ability and surface stability.

In short, Fe-based alloys offer a balance of cost, manufacturability, and performance at high temperatures. Their properties can be tailored via alloy design (Cr, Ni, Mo, and Al, etc.), processing innovation (AM), microstructural engineering (precipitates, grain control), and coating treatment. For ultra-high temperatures (>1000 °C), Ni/Co superalloys or ceramics may be required, but advanced Fe-based alloys (e.g., ODS, high-entropy alloys) continue to push boundaries. Further, the Fe-based alloys play a crucial role across various fields [17,21,22,23,24,25,26,27,28,29], due to their strength, corrosion resistance, and functional properties. In structural and mechanical applications, they are essential in construction, automotive, and aerospace industries, offering durability and toughness. Their high-temperature resistance makes them suitable for use in gas turbines, jet engines, and power plant components. Furthermore, the addition of alloying elements is intended not only to improve their high-temperature properties, but also to endow them with or enhance their functional applications, such as in the functional gradient Fe-based alloy [142], biodegradable Fe-based alloys [25,320], antibacterial alloy [321], Fe-based shape memory alloy [11], iron-based superconducting alloy [322,323], iron-based magnetic alloy (amorphous alloys [44], atypical Nd_2_Fe_14_B magnet [324,325], and others [326]), etc. [313,327,328]. 

## 6. Conclusions and Prospect

Fe-based high-temperature alloys have emerged as a vital materials class, offering an optimal balance of mechanical properties, thermal stability, oxidation resistance, and cost efficiency. While they may not match the peak performance of nickel-based superalloys in the most extreme conditions, their adaptable properties and economic advantages have secured their position across diverse industrial applications. Recent breakthroughs in alloy design, processing methods, and surface treatments are continuously pushing the boundaries of their high-temperature capabilities, opening new avenues for performance enhancement and application-specific optimization.

The field is currently witnessing transformative developments through several key approaches. Advanced alloying strategies, particularly those incorporating high-entropy alloy concepts, are creating new compositional spaces with enhanced properties. Simultaneously, the development of nanostructured and nanoprecipitate-strengthened variants is addressing longstanding challenges in creep resistance and oxidation performance. Modern manufacturing technologies, especially additive manufacturing, are revolutionizing component production by enabling complex geometries with precisely controlled microstructures. Perhaps most significantly, the integration of artificial intelligence and machine learning is accelerating every aspect of materials development, from initial discovery through performance prediction to process optimization.

However, several critical challenges must be overcome to fully realize this potential. Technical hurdles such as additive manufacturing defects, the high costs associated with some advanced alloys, and the need for sustainable production methods remain significant barriers. The implementation of AI/ML approaches faces its own challenges, including data limitations and interpretability issues. Looking ahead, the most promising opportunities lie at the intersection of these technologies—where computational design, nanoscale engineering, and advanced manufacturing converge. This synergistic approach promises to unlock new generations of Fe-based alloys capable of meeting the demands of tomorrow’s most challenging applications, from next-generation power systems to hypersonic platforms.

This review provides a comprehensive examination of contemporary developments in Fe-based high-temperature alloys, focusing on three critical aspects: innovative alloy design strategies, advanced processing techniques, and their resulting high-temperature performance characteristics. By synthesizing current knowledge and identifying future directions, it aims to provide both a reference for materials researchers and a roadmap for continued advancement in this essential field.

## Data Availability

No new data were created or analyzed in this study. Data sharing is not applicable to this article.

## References

[1] Stringer J., Wright I.G. (1995). Current Limitations of High-Temperature Alloys in Practical Applications. Oxid. Met..

[2] Hart G.L.W., Mueller T., Toher C., Curtarolo S. (2021). Machine Learning for Alloys. Nat. Rev. Mater..

[3] Sharma P., Tucker W.C., Balasubramanian G. (2025). Optimal Interplay of Charge Localization, Lattice Dynamics and Slip Systems Drives Structural Softening in Dilute W Alloys with Re Additives. Int. J. Refract. Met. Hard Mater..

[4] Meetham G.W. (1991). High-Temperature Materials—A General Review. J. Mater. Sci..

[5] Eswarappa Prameela S., Pollock T.M., Raabe D., Meyers M.A., Aitkaliyeva A., Chintersingh K.-L., Cordero Z.C., Graham-Brady L. (2022). Materials for Extreme Environments. Nat. Rev. Mater..

[6] Deevi S.C. (2021). Advanced Intermetallic Iron Aluminide Coatings for High Temperature Applications. Prog. Mater. Sci..

[7] Yu J., Lai H., Shi R., Peng Y., Miao L. (2023). Review of SiGe Alloys: Latest Research Progress and Optimization Strategies for Thermoelectric Properties. Adv. Ceram..

[8] Zhang W.T., Wang X.Q., Zhang F.Q., Cui X.Y., Fan B.B., Guo J.M., Guo Z.M., Huang R., Huang W., Li X.B. (2024). Frontiers in High Entropy Alloys and High Entropy Functional Materials. Rare Met..

[9] Qiu H.-P., Guan X.-Y., Zhao Y.-L., Yan J., Chen Y., Zhang Q.-Y., Zhang B.-Y., Chen M.-W. (2023). Influence of Matrix Modification on the Oxidation Resistance of SiC/SiC Composites. Adv. Ceram..

[10] Zhao Z., Liao W., Chen J., Jiao J., Wu C., Gou Y. (2024). Advanced Research on the Preparation and Application of Carbide Ceramic Fibers. J. Adv. Ceram..

[11] Xia J., Noguchi Y., Xu X., Odaira T., Kimura Y., Nagasako M., Omori T., Kainuma R. (2020). Iron-Based Superelastic Alloys with near-Constant Critical Stress Temperature Dependence. Science.

[12] Sun X., Song W., Liang J., Li J., Zhou Y. (2021). Research and Development in Materials and Processes of Superalloy Fabricated by Laser Additive Manufacturing. Jinshu Xuebao/Acta Metall. Sin..

[13] Darolia R. (2019). Development of Strong, Oxidation and Corrosion Resistant Nickel-Based Superalloys: Critical Review of Challenges, Progress and Prospects. Int. Mater. Rev..

[14] Sato J., Omori T., Oikawa K., Ohnuma I., Kainuma R., Ishida K. (2006). Cobalt-Base High-Temperature Alloys. Science.

[15] Su J.L., Jiang F.L., Teng J., Chen L.Q., Requena G., Yan M., Zhang L.C., Wang Y.M., Okulov I.V., Zhu H.M. (2024). Laser Additive Manufacturing of Titanium Alloys: Process, Materials and Post-Processing. Rare Met..

[16] Kollová A., Pauerová K. (2022). Superalloys—Characterization, Usage and Recycling. Manuf. Technol..

[17] Akande I.G., Oluwole O.O., Fayomi O.S.I., Odunlami O.A. (2020). Overview of Mechanical, Microstructural, Oxidation Properties and High-Temperature Applications of Superalloys. Mater. Today Proc..

[18] Sinha M.K., Pal A., Kishore K., Singh A., Archana, Sansanwal H., Sharma P. (2023). Applications of Sustainable Techniques in Machinability Improvement of Superalloys: A Comprehensive Review. Int. J. Interact. Des. Manuf..

[19] Pollock T.M. (2016). Alloy Design for Aircraft Engines. Nat. Mater..

[20] Xue Y., Zhu D., Pan J., You Z., Lyu X. (2024). Preparing Fe-Cr-Ni Alloy by Utilization of Limonitic Nickel Laterite Sinter. J. Cent. South Univ..

[21] Li Z., Zhang Y., Dong K., Zhang Z. (2022). Research Progress of Fe-Based Superelastic Alloys. Crystals.

[22] Wang X., Zhang Y., Zhang Z., Liu L., Wang B., Cui Y., Baker I., Cheng J. (2024). Development of Fe-Ni-Co-Al-Based Superelastic Alloys. Crit. Rev. Solid State Mater. Sci..

[23] Jiang G., Xu D., Feng P., Guo S., Yang J., Li Y. (2021). Corrosion of FeCrAl Alloys Used as Fuel Cladding in Nuclear Reactors. J. Alloys Compd..

[24] Pauletto G., Vaccari A., Groppi G., Bricaud L., Benito P., Boffito D.C., Lercher J.A., Patience G.S. (2020). FeCrAl as a Catalyst Support. Chem. Rev..

[25] Gorejová R., Haverová L., Oriňaková R., Oriňak A., Oriňak M. (2019). Recent Advancements in Fe-Based Biodegradable Materials for Bone Repair. J. Mater. Sci..

[26] Qi X., You J., Zhou J., Qiu K., Cui X., Tian J., Li B. (2023). A Review of Fe-Based Amorphous and Nanocrystalline Alloys: Preparations, Applications, and Effects of Alloying Elements. Phys. Status Solidi (A) Appl. Mater. Sci..

[27] Zhou H., Li L., Zhao Y., Shen M., Zhao H., Xiao Y., Liu S. (2025). Review of Rare Earth Oxide Doping-Modified Laser Cladding of Fe-Based Alloy Coatings. China Foundry.

[28] Del Olmo R., Tynkevych O., Łazińska M., Syrek K., Durejko T., Czerwiński M., Zaraska L., Tiwari R., Michalska-Domańska M. (2025). Anodizing of Iron-Based Alloys: Fundamentals, Recent Progress, and Applications. Rep. Prog. Phys..

[29] Cui J., Sun Y., Shi K., Deng S., Ma T., Du Y., Zhang J., Cheng N., Yuan X., He L. (2024). Invar Effect in the Wide and Higher Temperature Range by Coherent Coupling in Fe-Based Alloy. Adv. Funct. Mater..

[30] Vukelic G., Mihaljec B., Ivošević Š. (2025). Marine Environment Effect on Welded Additively Manufactured Stainless Steel AISI 316L. J. Mar. Sci. Eng..

[31] Ding P., Gong X., Sun L., Niu J., Zhang Y., Xu L. (2024). A Study on Crack Initiation and Propagation of Welded Joints under Explosive Load. J. Mar. Sci. Eng..

[32] Kishore K., Sarkar K., Arora K.S. (2023). Effect of Alloying Elements on Microstructure, Wear, and Corrosion Behavior of Fe-Based Hardfacing. Weld. World.

[33] Zhao L., Zhai G., Wu J., Chen X., Zhai Q. (2025). Microstructure and Mechanical Properties of a Novel Nb–V–Ce Multi-Microalloyed Low-Alloy Cast Steel. J. Iron Steel Res. Int..

[34] Xie A., Chen S., Wu Y., Jiang H., Rong L. (2022). Homogenization Temperature Dependent Microstructural Evolution and Mechanical Properties in a Nb-Stabilized Cast Austenitic Stainless Steel. Mater. Charact..

[35] Yang Y., Zhang C., Li J., Gou J., Ding Z. (2024). The Influence of Solute Elements on Grain Size, Hardness and Irradiation-Induced Defect Clusters in Dilute Fe-Based Alloys. J. Nucl. Mater..

[36] Qi Y., Ma X., Jiang Z., Ma L., Wang Z., Zhou C., Hasan M., Dobrzański L.A., Zhao J. (2025). Numerical and Experimental Investigation on the Formability of Stainless Steel-Copper Composites during Micro Deep Drawing. J. Cent. South Univ..

[37] Toozandehjani M., Moozarm Nia P., Abouzari Lotf E., Ostovan F., Shamshirsaz M. (2024). Aluminum Composite Powder as an Additive in Epoxy Coatings for Enhancement of Corrosion Protection of Carbon Steel. J. Cent. South Univ..

[38] Li X., Zhu G., Ma W., Shao R., Zhang Y., Zheng X. (2024). Crack Generation, Propagation Mechanism and Thermal Property of Zn-Coated Hot Stamping Steel. J. Cent. South Univ..

[39] Yao K., Dong Y., Jiang Z., Wang Y., Sun J. (2023). Effect of Cerium Addition on Cleanliness and Magnetic Properties of Fe-80Ni Permalloy. J. Cent. South Univ..

[40] Li J., Li B., Li P.-T., Zhang N., Shang H.-S. (2025). A ZnFeNiCoCr High-Entropy Alloy for Efficient Bifunctional Oxygen Electrocatalysis. Rare Met..

[41] Zhan Y., Cui Y., Wang J., Yu Z. (2024). Nano-Silica Modified Stainless Steel Mesh for Oil-Water Separation. Fine Chem..

[42] Chen R.Y., Yuen W.Y.D. (2003). Review of the High-Temperature Oxidation of Iron and Carbon Steels in Air or Oxygen. Oxid. Met..

[43] Inoue A. (2001). Bulk Amorphous and Nanocrystalline Alloys with High Functional Properties. Mater. Sci. Eng. A.

[44] Yang S., Zang B., Xiang M., Shen F., Song L., Gao M., Zhang Y., Huo J., Wang J. (2025). Designing Fe-Based Amorphous Alloys With Both Ultra-High Magnetization and Ultra-Low Coercivity Through Artificial Intelligence. Adv. Funct. Mater..

[45] Jia Z., Kang J., Zhang W.C., Wang W.M., Yang C., Sun H., Habibi D., Zhang L.C. (2017). Surface Aging Behaviour of Fe-Based Amorphous Alloys as Catalysts during Heterogeneous Photo Fenton-like Process for Water Treatment. Appl. Catal. B Environ..

[46] Ani M.H.B., Kodama T., Ueda M., Kawamura K., Maruyama T. (2009). The Effect of Water Vapor on High Temperature Oxidation of Fe-Cr Alloys at 1073 K. Mater. Trans..

[47] Li Y.S., Niu Y., Spiegel M. (2007). High Temperature Interaction of Al/Si-Modified Fe-Cr Alloys with KCl. Corros. Sci..

[48] Biswas S., Ramachandra S., Hans P., Kumar S.P.S. (2022). Materials for Gas Turbine Engines: Present Status, Future Trends and Indigenous Efforts. J. Indian Inst. Sci..

[49] Airiskallio E., Nurmi E., Heinonen M.H., Väyrynen I.J., Kokko K., Ropo M., Punkkinen M.P.J., Pitkänen H., Alatalo M., Kollár J. (2010). High Temperature Oxidation of Fe-Al and Fe-Cr-Al Alloys: The Role of Cr as a Chemically Active Element. Corros. Sci..

[50] Felten E.J. (1961). High-Temperature Oxidation of Fe-Cr Base Alloys with Particular Reference to Fe-Cr-Y Alloys. J. Electrochem. Soc..

[51] Tedmon C.S. (1967). The High-Temperature Oxidation of Fe-Cr Alloys in the Composition Range of 25–95% Cr. J. Electrochem. Soc..

[52] Qiao Y., Wang P., Qi W., Du S., Liu Z., Meng F., Zhang X., Wang K., Li Q., Yao Z. (2020). Mechanism of Al on FeCrAl Steam Oxidation Behavior and Molecular Dynamics Simulations. J. Alloys Compd..

[53] Babu N., Balasubramaniam R., Ghosh A. (2001). High-Temperature Oxidation of Fe3Al-Based Iron Aluminides in Oxygen. Corros. Sci..

[54] Morris D.G., Muñoz-Morris M.A., Baudin C. (2004). The High-Temperature Strength of Some Fe3Al Alloys. Acta Mater..

[55] Tortorelli P.F., Natesan K. (1998). Critical Factors Affecting the High-Temperature Corrosion Performance of Iron Aluminides. Mater. Sci. Eng. A.

[56] Fischer R.A., Campbell A.J., Caracas R., Reaman D.M., Heinz D.L., Dera P., Prakapenka V.B. (2014). Equations of State in the Fe-FeSi System at High Pressures and Temperatures. AGU J. Geophys. Res. Solid Earth.

[57] Zhang Y., Luo K., Hou M., Driscoll P., Salke N.P., Greenberg E., Hemley R.J., Cohen R.E., Lin J. (2022). Thermal Conductivity of Fe-Si Alloys and Thermal Stratification in Earth’s Core. Proc. Natl. Acad. Sci. USA.

[58] Li W., Lian J., Wang D., Zhang S., Han C., Du Z., Li F. (2024). Effect of Si Addition on Structure and Corrosion Resistance of FeCoNiCr High-Entropy Alloy Coating. Materials.

[59] Mao W.L., Campbell A.J., Heinz D.L., Shen G. (2006). Phase Relations of Fe-Ni Alloys at High Pressure and Temperature. Phys. Earth Planet. Inter..

[60] Liu N., Cao X., Zhao T., Zhang Z.W. (2021). Progress of Zirconium Alloying in Iron-Based Alloys and Steels. Mater. Sci. Technol..

[61] Qi W., Yang K., Wang P., Du S., Bai C., Wang X., Qiao Y., Zheng T., Zhang L.L., Zhang X. (2022). High-Temperature Steam Oxidation Behavior of an FeCrAl Alloy with Controlled Addition of Mo. J. Mater. Sci..

[62] Dong X., Saksena A., Tehranchi A., Gault B., Ponge D., Sun B., Raabe D. (2025). Interfacial Boron Segregation in a High-Mn and High-Al Multiphase Lightweight Steel. Acta Mater..

[63] Wen Y.R., Li Y.P., Hirata A., Zhang Y., Fujita T., Furuhara T., Liu C.T., Chiba A., Chen M.W. (2013). Synergistic Alloying Effect on Microstructural Evolution and Mechanical Properties of Cu Precipitation-Strengthened Ferritic Alloys. Acta Mater..

[64] Albrecht A., Song S.Y., Yoo S., Lee C., Krämer M., Hans M., Gault B., Ma Y., Raabe D., Sohn S.S. (2025). An Atomic-Scale View at γ’-Fe_4_N as Hydrogen Barrier Material. Adv. Mater. Interfaces.

[65] Steiner T., Mittemeijer E.J. (2016). Alloying Element Nitride Development in Ferritic Fe-Based Materials Upon Nitriding: A Review. J. Mater. Eng. Perform..

[66] Du J.L., Feng Y.L., Zhang M. (2021). Construction of a Machine-Learning-Based Prediction Model for Mechanical Properties of Ultra-Fine-Grained Fe–C Alloy. J. Mater. Res. Technol..

[67] Shao L. (2024). Effect of Carbon on Void Nucleation in Iron. Materials.

[68] Risal S., Singh N., Yao Y., Sun L., Risal S., Zhu W. (2024). Accelerating Elastic Property Prediction in Fe-C Alloys through Coupling of Molecular Dynamics and Machine Learning. Materials.

[69] Akca E., Gürsel A. (2015). A Review on Superalloys and IN718 Nickel-Based INCONEL Superalloy. PEN.

[70] Fang W., Huang J., Peng T., Long Y., Yin F. (2024). Machine Learning-Based Performance Predictions for Steels Considering Manufacturing Process Parameters: A Review. J. Iron Steel Res. Int..

[71] Cemernek D., Cemernek S., Gursch H., Pandeshwar A., Leitner T., Berger M., Klösch G., Kern R. (2022). Machine Learning in Continuous Casting of Steel: A State-of-the-Art Survey. J. Intell. Manuf..

[72] Pan G., Wang F., Shang C., Wu H., Wu G., Gao J., Wang S., Gao Z., Zhou X., Mao X. (2023). Advances in Machine Learning- and Artificial Intelligence-Assisted Material Design of Steels. Int. J. Miner. Metall. Mater..

[73] Guo S., Yu J., Liu X., Wang C., Jiang Q. (2019). A Predicting Model for Properties of Steel Using the Industrial Big Data Based on Machine Learning. Comput. Mater. Sci..

[74] Shen C., Wang C., Wei X., Li Y., Van Der Zwaag S., Xu W. (2019). Physical Metallurgy-Guided Machine Learning and Artificial Intelligent Design of Ultrahigh-Strength Stainless Steel. Acta Mater..

[75] Winkelmann H., Badisch E., Kirchgaßner M., Danninger H. (2009). Wear Mechanisms at High Temperatures. Part 1: Wear Mechanisms of Different Fe-Based aAlloys at Elevated Temperatures. Tribol. Lett..

[76] Torres H., Varga M., Ripoll M.R. (2016). High Temperature Hardness of Steels and Iron-Based Alloys. Mater. Sci. Eng. A.

[77] Qi W., Qiao Y., Ru W., Wang X., Zhang X., Zheng T., Du S., Wang P., Yang K. (2024). High-Temperature Steam Oxidation and Surface Microstructure Evolution of Fe13Cr6Al(1–4)Mo0.15Y Alloys. Metals.

[78] Xu K.D., Ren Z.M., Li C.J. (2014). Progress in Application of Rare Metals in Superalloys. Rare Met..

[79] Lee H., Son H., Choi-Yim H. (2024). Effect of Nb Addition and Annealing Treatment on Structural and Magnetic Properties of Fe–Si–B–P–Cu Alloy Ribbons. Intermetallics.

[80] Ni Y., Qi W., Zhao L., Li D., Qiao Y., Zhou J., Wang P., Yang K. (2025). Thermo-Mechanical Properties and Oxidation Behavior of FeCrAl Alloys with Si and Y Addition. Metals.

[81] Ni Y., Zhou J., Zhao L., Li D., Qiao Y., Yang K., Wang X., Bai C., Wang P., Li X. (2025). A First-Principle Study of the Effect of Yttrium on the Oxidation Resistance of FeCrAl Alloys. Mater. Technol..

[82] Sohail Y., Zhang C., Gao S., Zhang J., Song W., Li X., Wang B., Li S., Xue D., Liu G. (2024). A Complex Concentrated Alloy with Record-High Strength-Toughness at 77 K. Adv. Mater..

[83] Chu Q., Yang D., Su C., Li J., Cao K., Xie W., Yan F., Yan C., Qin S. (2024). Effect of Multi-Element Synergistic Addition on the Microstructure Evolution and Performance Enhancement of Laser Hot-Wire Cladded Fe-Based Alloy. Int. J. Press. Vessel. Pip..

[84] Persdotter A., Boll T., Ssenteza V., Jonsson T. (2024). Insight into the Influence of Alloying Elements on the Secondary Corrosion Protection of Fe-Base Alloys by Means of Atom Probe Tomography. Corros. Sci..

[85] Terrani K.A. (2018). Accident Tolerant Fuel Cladding Development: Promise, Status, and Challenges. J. Nucl. Mater..

[86] Wei L.L., Wang Y.G., Misra R.D.K., Chen J. (2024). Understanding the High-Temperature Oxidation Resistance of Heat-Resistant Austenitic Stainless Steel with Gradient Nanostructure. Corros. Sci..

[87] Wang P., Qi W., Yang K., Qiao Y., Wang X., Zheng T., Bai C., Liu Z., Zhang X. (2022). Systematic Investigation of the Oxidation Behavior of Fe-Cr-Al Cladding Alloys in High-Temperature Steam. Corros. Sci..

[88] Badini C., Laurella F. (2001). Oxidation of FeCrAl Alloy: Influence of Temperature and Atmosphere on Scale Growth Rate and Mechanism. Surf. Coat. Technol..

[89] Gussev M.N., Field K.G., Yamamoto Y. (2017). Design, Properties, and Weldability of Advanced Oxidation-Resistant FeCrAl Alloys. Mater. Des..

[90] Yamamoto Y., Pint B.A., Terrani K.A., Field K.G., Yang Y., Snead L.L. (2015). Development and Property Evaluation of Nuclear Grade Wrought FeCrAl Fuel Cladding for Light Water Reactors. J. Nucl. Mater..

[91] Qian L., Liu Y., Huang T., Chen W., Du S., Yin C., Xiong Q. (2023). Research Progress in High-Temperature Thermo-Mechanical Behavior for Modelling FeCrAl Cladding under Loss-of-Coolant Accident Condition. Prog. Nucl. Energy.

[92] Palaniappan S., Joshi S.S., Sharma S., Radhakrishnan M., Krishna K.V.M., Dahotre N.B. (2024). Additive Manufacturing of FeCrAl Alloys for Nuclear Applications—A Focused Review. Nucl. Mater. Energy.

[93] Magalhães I.R., Chaves L.V.G., de Castro V.F., Reis P.A.L., Costa A.L., Veloso M.A.F., Pereira C. (2024). Comparative Analysis of Different FeCrAl Alloys in Pressurized Water Reactors. Nucl. Eng. Des..

[94] Lindmark H., Phother J., Dolores Paz Olausson M., Nockert J., Lind F., Jonasson A., Barišić V., Vänskä K., Rioja-Monllor L., Liske J. (2023). A Material Degradation Study of Novel FeCrAl Alloys, Stainless Steels and Nickel Base Alloy in Fluidized Bed Heat Exchangers of a Waste-Fired CFB Boiler. Fuel.

[95] Niu M.C., Yin L.C., Yang K., Luan J.H., Wang W., Jiao Z.B. (2021). Synergistic Alloying Effects on Nanoscale Precipitation and Mechanical Properties of Ultrahigh-Strength Steels Strengthened by Ni3Ti, Mo-Enriched, and Cr-Rich Co-Precipitates. Acta Mater..

[96] Gao J., Jiang S., Zhang H., Huang Y., Guan D., Xu Y., Guan S., Bendersky L.A., Davydov A.V., Wu Y. (2021). Facile Route to Bulk Ultrafine-Grain Steels for High Strength and Ductility. Nature.

[97] Xu K., An J., Zhang L., Bai C., Tu J., Luo J. (2023). Composition Optimization of Cobalt-Free Fe-Cr-Ni-Al/Ti Multi-Principal Element Alloys for Strength-Ductility Trade-off Based on Machine Learning. Mater. Today Commun..

[98] Baik S., Wang S.Y., Liaw P.K., Dunand D.C. (2018). Increasing the Creep Resistance of Fe-Ni-Al-Cr Superalloys via Ti Additions by Optimizing the B2/L21 Ratio in Composite Nano-Precipitates. Acta Mater..

[99] Cho K., Ikeda K., Yasuda H.Y. (2018). Improvement of Room and High Temperature Tensile Properties of NiAl-Strengthened Ferritic Heat-Resistant Steels through Mo Addition. Mater. Sci. Eng. A.

[100] Sundar R.S., Deevi S.C. (2003). High-Temperature Strength and Creep Resistance of FeAl. Mater. Sci. Eng. A.

[101] Show B.K., Veerababu R., Balamuralikrishnan R., Malakondaiah G. (2010). Effect of Vanadium and Titanium Modification on the Microstructure and Mechanical Properties of a Microalloyed HSLA Steel. Mater. Sci. Eng. A.

[102] Jiang S., Wang H., Wu Y., Liu X., Chen H., Yao M., Gault B., Ponge D., Raabe D., Hirata A. (2017). Ultrastrong Steel via Minimal Lattice Misfit and High-Density Nanoprecipitation. Nature.

[103] Song G., Hong S.J., Lee J.K., Song S.H., Hong S.H., Kim K.B., Liaw P.K. (2019). Optimization of B2/L21 Hierarchical Precipitate Structure to Improve Creep Resistance of a Ferritic Fe-Ni-Al-Cr-Ti Superalloy via Thermal Treatments. Scr. Mater..

[104] Park K., Cho B., Hong S.J., Lim K.R., Lee C., Song G. (2022). Outstanding High-Temperature Strength of Novel Fe–Cr–Ni–Al–V Ferritic Alloys with Hierarchical B2–NiAl Precipitates. Mater. Sci. Eng. A.

[105] Qian F., Sharp J., Rainforth W.M. (2016). Characterisation of L21-Ordered Ni2TiAl Precipitates in [Formula Presented] Maraging Steels. Mater. Charact..

[106] Song G., Sun Z., Clausen B., Liaw P.K. (2017). Microstructural Characteristics of a Ni_2_TiAl-Precipitate-Strengthened Ferritic Alloy. J. Alloys Compd..

[107] Wang Z., Fan M., Zhang Y., Li J., Liu L., Han J., Li X., Zhang Z. (2024). A Novel Immiscible High Entropy Alloy Strengthened via L12-Nanoprecipitate. J. Cent. South Univ..

[108] Yasuda H.Y., Odawara Y., Soma K., Yoshimoto T., Cho K. (2017). Effects of CoAl Precipitates on Deformation Behavior of Fe-Al-Co Single Crystals. Intermetallics.

[109] Chen N., Chen L.W., Teng H., Li Z.Y., Yuan T.C. (2024). A Modified M2 High-Speed Steel Enhanced by in-Situ Synthesized Core-Shell MC Carbides. J. Cent. South Univ..

[110] Zhang Z., Liu C.T., Miller M.K., Wang X.L., Wen Y., Fujita T., Hirata A., Chen M., Chen G., Chin B.A. (2013). A Nanoscale Co-Precipitation Approach for Property Enhancement of Fe-Base Alloys. Sci. Rep..

[111] Jiao Z.B., Luan J.H., Miller M.K., Yu C.Y., Liu C.T. (2016). Group Precipitation and Age Hardening of Nanostructured Fe-Based Alloys with Ultra-High Strengths. Sci. Rep..

[112] Teng Z.K., Zhang F., Miller M.K., Liu C.T., Huang S., Chou Y.T., Tien R.H., Chang Y.A., Liaw P.K. (2012). New NiAl-Strengthened Ferritic Steels with Balanced Creep Resistance and Ductility Designed by Coupling Thermodynamic Calculations with Focused Experiments. Intermetallics.

[113] Jain R., Jain S., Nagarjuna C., Samal S., Rananavare A.P., Dewangan S.K., Ahn B. (2025). A Comprehensive Review on Hot Deformation Behavior of High-Entropy Alloys for High Temperature Applications. Met. Mater. Int..

[114] Dewangan S.K., Mangish A., Kumar S., Sharma A., Ahn B., Kumar V. (2022). A Review on High-Temperature Applicability: A Milestone for High Entropy Alloys. Eng. Sci. Technol. Int. J..

[115] Liang J., Cao G., Zeng M., Fu L. (2024). Controllable Synthesis of High-Entropy Alloys. Chem. Soc. Rev..

[116] Hsu W.-L., Tsai C.-W., Yeh A.-C., Yeh J.-W. (2024). Clarifying the Four Core Effects of High-Entropy Materials. Nat. Rev. Chem..

[117] George E.P., Raabe D., Ritchie R.O. (2019). High-Entropy Alloys. Nat. Rev. Mater..

[118] Praveen S., Kim H.S. (2018). High-Entropy Alloys: Potential Candidates for High-Temperature Applications—An Overview. Adv. Eng. Mater..

[119] Rao Z., Tung P., Xie R., Wei Y., Zhang H., Ferrari A., Klaver T.P.C., Körmann F., Sukumar P.T., da Silva A.K. (2022). Machine Learning-Enabled High-Entropy Alloy Discovery. Science.

[120] Chen J., Zhou X., Wang W., Liu B., Lv Y., Yang W., Xu D., Liu Y. (2018). A Review on Fundamental of High Entropy Alloys with Promising High–Temperature Properties. J. Alloys Compd..

[121] Kim L., Scougale W.R., Sharma P., Shirato N., Wieghold S., Rose V., Chen W., Balasubramanian G., Chien T. (2024). Distinguishing Elements at the Sub-Nanometer Scale on the Surface of a High Entropy Alloy. Adv. Mater..

[122] Miracle D.B., Senkov O.N. (2017). A Critical Review of High Entropy Alloys and Related Concepts. Acta Mater..

[123] Zhu J., Lu S., Jin Y., Xu L., Xu X., Yin C., Jia Y. (2020). High-Temperature Oxidation Behaviours of AlCoCrFeNi High-Entropy Alloy at 1073–1273 K. Oxid. Met..

[124] Tokarewicz M., Grądzka-Dahlke M. (2021). Review of Recent Research on Alcocrfeni High-Entropy Alloy. Metals.

[125] Munitz A., Salhov S., Hayun S., Frage N. (2016). Heat Treatment Impacts the Micro-Structure and Mechanical Properties of AlCoCrFeNi High Entropy Alloy. J. Alloys Compd..

[126] Tsao T., Chang Y., Chang K., Yeh J., Chiou M., Jian S., Kuo C., Wang W., Murakami H. (2015). Developing New Type of High Temperature Alloys–High Entropy Superalloys. Int. J. Metall. Mater. Eng..

[127] Zhang K., Zhang X., Zhang E., Wei R., Wang L., Chen J., Yuan S., Han Z., Chen C., Li F. (2021). Strengthening of Ferrous Medium Entropy Alloys by Promoting Phase Transformation. Intermetallics.

[128] Seol J.B., Bae J.W., Li Z., Chan Han J., Kim J.G., Raabe D., Kim H.S. (2018). Boron Doped Ultrastrong and Ductile High-Entropy Alloys. Acta Mater..

[129] Wei R., Zhang K., Chen L., Han Z., Wang T., Chen C., Jiang J., Hu T., Li F. (2020). Novel Co-Free High Performance TRIP and TWIP Medium-Entropy Alloys at Cryogenic Temperatures. J. Mater. Sci. Technol..

[130] Wang Z., Genc A., Baker I. (2017). Direct versus Indirect Particle Strengthening in a Strong, Ductile FeNiMnAlTi High Entropy Alloy. Mater. Charact..

[131] Qin M., Jin X., Zhang M., Yang H., Qiao J. (2021). Twinning Induced Remarkable Strain Hardening in a Novel Fe50Mn20Cr20Ni10 Medium Entropy Alloy. J. Iron Steel Res. Int..

[132] Feng R., Zhang C., Gao M.C., Pei Z., Zhang F., Chen Y., Ma D., An K., Poplawsky J.D., Ouyang L. (2021). High-Throughput Design of High-Performance Lightweight High-Entropy Alloys. Nat. Commun..

[133] Tsianikas S.J., Chen Y., Jeong J., Zhang S., Xie Z. (2022). Forging Strength–Ductility Unity in a High Entropy Steel. J. Mater. Sci. Technol..

[134] Mitrica D., Badea I.C., Serban B.A., Olaru M.T., Vonica D., Burada M., Piticescu R.-R., Popov V.V. (2021). Complex Concentrated Alloys for Substitution of Critical Raw Materials in Applications for Extreme Conditions. Materials.

[135] Qin S., Zhu Z., Ma H., Wang G., Zhou Y. (2023). Effect of Retained Austenite on the Fatigue Performance of Novel High Carbon Quenching-Partitioning-Tempering Steel. J. Cent. South Univ..

[136] Shaik K.N.R., Bortolotti M., Leizaola I., Lagos Gomez M.A., Menapace C. (2024). Production and Characterization of Fine-Grained Multielement AlCoxCrFeNi (x = 1, 0.75, 0.5) Alloys for High-Temperature Applications. Materials.

[137] Lu Y., Dong Y., Guo S., Jiang L., Kang H., Wang T., Wen B., Wang Z., Jie J., Cao Z. (2014). A Promising New Class of High-Temperature Alloys: Eutectic High-Entropy Alloys. Sci. Rep..

[138] Yang C. (2024). Effect of Warm Rolling on Microstructure and Mechanical Properties of Fe_50_Mn_30_Co_10_Cr_10_ High-Entropy Alloy. J. Cent. South Univ..

[139] Stallybrass C., Sauthoff G. (2004). Ferritic Fe-Al-Ni-Cr Alloys with Coherent Precipitates for High-Temperature Applications. Mater. Sci. Eng. A.

[140] Nene S.S., Liu K., Frank M., Mishra R.S., Brennan R.E., Cho K.C., Li Z., Raabe D. (2017). Enhanced Strength and Ductility in a Friction Stir Processing Engineered Dual Phase High Entropy Alloy. Sci. Rep..

[141] Kartikeya Sarma I., Selvaraj N., Kumar A. (2023). Parametric Investigation and Characterization of 17-4 PH Stainless Steel Parts Fabricated by Selective Laser Melting. J. Cent. South Univ..

[142] Ma Z., Liu W., Li W., Liu H., Song J., Liu Y., Huang Y., Xia Y., Wang Z., Liu B. (2024). Additive Manufacturing of Functional Gradient Materials: A Review of Research Progress and Challenges. J. Alloys Compd..

[143] Schafrik R., Sprague R. (2008). Superalloy Technology—A Perspective on Critical Innovations for Turbine Engines. Key Eng. Mater..

[144] Sun F., Xu H., Meng Y., Lu Z., Chen S., Wei Q., Bai C. (2023). BERT and Pareto Dominance Applied to Biological Strategy Decision for Bio-Inspired Design. Adv. Eng. Inform..

[145] Pan Q., Ding K., Guo S., Lu N., Tao N., Zhu T., Lu L. (2025). Superior Resistance to Cyclic Creep in a Gradient Structured Steel. Science.

[146] Xu H., Lu Z., Ukai S., Oono N., Liu C. (2017). Effects of Annealing Temperature on Nanoscale Particles in Oxide Dispersion Strengthened Fe-15Cr Alloy Powders with Ti and Zr Additions. J. Alloys Compd..

[147] Wang B., Zhu D., Zhang C., Zhou X., Wu H., Wang S., Wu G., Gao J., Zhao H., Mao X. (2024). Influence of Typical Elements and Heat Treatment Parameters on Hardenability in Steel: A Review. J. Iron Steel Res. Int..

[148] Rahman M.H., Rasel M.A.J., Smyth C.M., Waryoba D., Haque A. (2024). Radiation Damage Mitigation in FeCrAl Alloy at Sub-Recrystallization Temperatures. Materials.

[149] Wang Y., Wei L., Tan Z., Yang J., Yu C., Wang S., Yang L., Zheng H., Lei X., Misra R.D.K. (2025). Unveiling the Relationship of Fatigue Behavior with the Microstructure of 321 Stainless Steel with Gradient Structure at 550 °C. Materialia.

[150] Liu B., Li J., Guo W., Xu P., Zhang S., Zhang Y. (2022). Progress in Corrosion-Resistant Coatings on Surface of Low Alloy Steel. J. Iron Steel Res. Int..

[151] Xavier J.R. (2024). Novel Multifunctional Epoxy Based Graphitic Carbon Nitride/Silanized TiO_2_ Nanocomposite as Protective Coatings for Steel Surface against Corrosion and Flame in the Shipping Industry. J. Cent. South Univ..

[152] Ban H., Shi G. (2018). A Review of Research on High-Strength Steel Structures. Proc. Inst. Civ. Eng.-Struct. Build..

[153] Curtze S., Kuokkala V.-T., Hokka M., Peura P. (2009). Deformation Behavior of TRIP and DP Steels in Tension at Different Temperatures over a Wide Range of Strain Rates. Mater. Sci. Eng. A.

[154] Han J., Nam J.-H., Lee Y.-K. (2016). The Mechanism of Hydrogen Embrittlement in Intercritically Annealed Medium Mn TRIP Steel. Acta Mater..

[155] Herrera C., Ponge D., Raabe D. (2011). Design of a Novel Mn-Based 1GPa Duplex Stainless TRIP Steel with 60% Ductility by a Reduction of Austenite Stability. Acta Mater..

[156] Bai Y., Momotani Y., Chen M.C., Shibata A., Tsuji N. (2016). Effect of Grain Refinement on Hydrogen Embrittlement Behaviors of High-Mn TWIP Steel. Mater. Sci. Eng. A.

[157] Raabe D., Sun B., Da Kwiatkowski Silva A., Gault B., Yen H.-W., Sedighiani K., Thoudden Sukumar P., Souza Filho I.R., Katnagallu S., Jägle E. (2020). Current Challenges and Opportunities in Microstructure-Related Properties of Advanced High-Strength Steels. Met. Mater. Trans. A.

[158] Liang W., Geng R., Zhi J., Li J., Huang F. (2022). Oxide Metallurgy Technology in High Strength Steel: A Review. Materials.

[159] Li K., Yang T., Gong N., Wu J., Wu X., Zhang D.Z., Murr L.E. (2023). Additive Manufacturing of Ultra-High Strength Steels: A Review. J. Alloys Compd..

[160] Soleimani M., Kalhor A., Mirzadeh H. (2020). Transformation-Induced Plasticity (TRIP) in Advanced Steels: A Review. Mater. Sci. Eng. A.

[161] Tan X., Ponge D., Lu W., Xu Y., He H., Yan J., Wu D., Raabe D. (2020). Joint Investigation of Strain Partitioning and Chemical Partitioning in Ferrite-Containing TRIP-Assisted Steels. Acta Mater..

[162] Lin Y., Yu W., Wang G., Li Z., Jiang Y., Feng J., Chong X. (2024). Exploring the Effect of Alloying Elements on the Thermoelasticity and Strength of Bcc Fe-Based Alloys by First-Principles Phonon Calculations. J. Mater. Res. Technol..

[163] Li T., Yang Z., Cui J., Chen W., Almatani R., Wu Y. (2025). Prediction and Optimization of Stretch Flangeability of Advanced High Strength Steels Utilizing Machine Learning Approaches. Sci. Rep..

[164] Zhang D., Sun S., Qiu D., Gibson M.A., Dargusch M.S., Brandt M., Qian M., Easton M. (2018). Metal Alloys for Fusion-Based Additive Manufacturing. Adv. Eng. Mater..

[165] Lewandowski J.J., Seifi M. (2016). Metal Additive Manufacturing: A Review of Mechanical Properties. Annu. Rev. Mater. Res..

[166] An D., Zhou Y., Liu X., Wang H., Li S., Xiao Y., Li R., Li X., Han X., Chen J. (2023). Exploring Structural Origins Responsible for the Exceptional Mechanical Property of Additively Manufactured 316L Stainless Steel via In-Situ and Comparative Investigations. Int. J. Plast..

[167] Jarlöv A., Zhu Z., Ji W., Gao S., Hu Z., Vivegananthan P., Tian Y., Kripalani D.R., Fan H., Seet H.L. (2024). Recent Progress in High-Entropy Alloys for Laser Powder Bed Fusion: Design, Processing, Microstructure, and Performance. Mater. Sci. Eng. R Rep..

[168] Sun F., Xu H., Zhang H., Bai C., Colombo P. (2023). Biomimetic Composite Structural Water Hydraulic Valve Plug for Erosive Wear Resistance Based on Additive Manufacturing Processes. J. Manuf. Process..

[169] Bajaj P., Hariharan A., Kini A., Kürnsteiner P., Raabe D., Jägle E.A. (2020). Steels in Additive Manufacturing: A Review of Their Microstructure and Properties. Mater. Sci. Eng. A.

[170] Tan Q., Chang H., Liang G., Luzin V., Yin Y., Wang F., Cheng X., Yan M., Zhu Q., Hutchinson C. (2024). High Performance Plain Carbon Steels Obtained through 3D-Printing. Nat. Commun..

[171] Roberts D., Zhang Y., Charit I., Zhang J. (2018). A Comparative Study of Microstructure and High-Temperature Mechanical Properties of 15-5 PH Stainless Steel Processed via Additive Manufacturing and Traditional Manufacturing. Prog. Addit. Manuf..

[172] AlMangour B., Grzesiak D., Yang J.-M. (2017). Scanning Strategies for Texture and Anisotropy Tailoring during Selective Laser Melting of TiC/316L Stainless Steel Nanocomposites. J. Alloys Compd..

[173] Prashanth K.G., Eckert J. (2017). Formation of Metastable Cellular Microstructures in Selective Laser Melted Alloys. J. Alloys Compd..

[174] Demirci S., Tünçay M.M. (2025). Growth Kinetics of the Borided 316L Stainless Steel Obtained by Selective Laser. J. Cent. South Univ..

[175] Lin Y.-T., Tsai M.-Y., Yen S.-Y., Lung G.-H., Yei J.-T., Hsu K.-J., Chen K.-J. (2024). Comparing the Performance of Rolled Steel and 3D-Printed 316L Stainless Steel. Micromachines.

[176] Liu J., Li C., Yang H., Liu J., Wang J., Deng L., Fang L., Yang C. (2024). Study on Laser-Electrochemical Hybrid Polishing of Selective Laser Melted 316L Stainless Steel. Micromachines.

[177] Zhang Z., Mativenga P., Zhang W., Huang S. (2024). Deep Learning-Driven Prediction of Mechanical Properties of 316L Stainless Steel Metallographic by Laser Powder Bed Fusion. Micromachines.

[178] Cai Z., Wang K., Zhu J., Jiao X., Du X., Wang H. (2024). Characterization of Microstructure and Texture of Lean Duplex Stainless Steel 2101 Produced by Underwater Laser Wire Direct Energy Deposition. J. Cent. South Univ..

[179] Caniou R., Rado C., Gaillard G.-C., Tosoni O., Flament C., Garandet J.-P. (2023). Influence of Process Parameters on the Microstructure of Laser Printed NdFeB Alloys. J. Magn. Magn. Mater..

[180] Wang H., Feng W., Liu D., Zhang G., Liu Y., Wang J., Zou L. (2023). Iron-Based Soft Magnetic Materials Fabricated by Laser Additive Manufacturing. Eng. Sci..

[181] Korkmaz M.E., Gupta M.K., Robak G., Moj K., Krolczyk G.M., Kuntoğlu M. (2022). Development of Lattice Structure with Selective Laser Melting Process: A State of the Art on Properties, Future Trends and Challenges. J. Manuf. Process..

[182] Bahrudin M.T.A.H., Januddi F.S., Sarian M.N., Arafat A., Nur H., Md Yusop A.H. (2025). Current Status of Additively Manufactured Porous Fe-Based Scaffolds for Bone Applications: A Review from Design, Corrosion and Biocompatibility Viewpoints. Mater. Today Commun..

[183] Limón I., Bedmar J., Fernández-Hernán J.P., Multigner M., Torres B., Rams J., Cifuentes S.C. (2024). A Review of Additive Manufacturing of Biodegradable Fe and Zn Alloys for Medical Implants Using Laser Powder Bed Fusion (LPBF). Materials.

[184] Sun Q., Tan X., Ding M., Cao B., Iwamoto T. (2024). A Review of Additively Manufactured Iron-Based Shape Memory Alloys. Crystals.

[185] Cheng L., Deng C., Huang Y., Li K., Han C. (2025). Mechanical and Catalytic Degradation Properties of Porous FeMnCoCr High-Entropy Alloy Structures Fabricated by Selective Laser Melting Additive Manufacturing. Materials.

[186] Raabe D. (2023). The Materials Science behind Sustainable Metals and Alloys. Chem. Rev..

[187] Rechberger K., Spanlang A., Sasiain Conde A., Wolfmeir H., Harris C. (2020). Green Hydrogen-Based Direct Reduction for Low-Carbon Steelmaking. Steel Res. Int..

[188] Wang R.R., Zhao Y.Q., Babich A., Senk D., Fan X.Y. (2021). Hydrogen Direct Reduction (H-DR) in Steel Industry—An Overview of Challenges and Opportunities. J. Clean. Prod..

[189] Wei S., Ma Y., Raabe D. (2024). One Step from Oxides to Sustainable Bulk Alloys. Nature.

[190] Wei S., Ma Y., Raabe D. (2024). Reactive Vapor-Phase Dealloying-Alloying Turns Oxides into Sustainable Bulk Nano-Structured Porous Alloys. Sci. Adv..

[191] Shang Y., Liu S., Liang Z., Pyczak F., Lei Z., Heidenreich T., Schökel A., Kai J., Gizer G., Dornheim M. (2022). Developing Sustainable FeTi Alloys for Hydrogen Storage by Recycling. Commun. Mater..

[192] Broadbent C. (2016). Steel’s Recyclability: Demonstrating the Benefits of Recycling Steel to Achieve a Circular Economy. Int. J. Life Cycle Assess..

[193] Han L., Mu W., Wei S., Liaw P.K., Raabe D. (2024). Sustainable High-Entropy Materials?. Sci. Adv..

[194] Svetlizky D. (2022). Laser-Based Directed Energy Deposition (DED-LB) of Advanced Materials. Mater. Sci..

[195] Heim J.W., Vander Wal R.L. (2023). NdFeB Permanent Magnet Uses, Projected Growth Rates and Nd Plus Dy Demands across End-Use Sectors through 2050: A Review. Minerals.

[196] Yang Y., Walton A., Sheridan R., Güth K., Gauß R., Gutfleisch O., Buchert M., Steenari B.-M., Van Gerven T., Jones P.T. (2017). REE Recovery from End-of-Life NdFeB Permanent Magnet Scrap: A Critical Review. J. Sustain. Metall..

[197] Kaya M. (2024). An Overview of NdFeB Magnets Recycling Technologies. Curr. Opin. Green Sustain. Chem..

[198] Kwiatkowski Da Silva A., Souza Filho I.R., Lu W., Zilnyk K.D., Hupalo M.F., Alves L.M., Ponge D., Gault B., Raabe D. (2022). A Sustainable Ultra-High Strength Fe18Mn3Ti Maraging Steel through Controlled Solute Segregation and α-Mn Nanoprecipitation. Nat. Commun..

[199] Xie Q., Suvarna M., Li J., Zhu X., Cai J., Wang X. (2021). Online Prediction of Mechanical Properties of Hot Rolled Steel Plate Using Machine Learning. Mater. Des..

[200] Bai X., Zhang X. (2025). Artificial Intelligence-Powered Materials Science. Nano-Micro Lett..

[201] Wen C., Zhang Y., Wang C., Huang H., Wu Y., Lookman T., Su Y. (2025). Machine-Learning-Assisted Compositional Design of Refractory High-Entropy Alloys with Optimal Strength and Ductility. Engineering.

[202] Liu Y., Zhao T., Ju W., Shi S. (2017). Materials Discovery and Design Using Machine Learning. J. Mater..

[203] Han Z., Xia S., Chen Z., Guo Y., Li Z., Huang Q., Liu X.-J., Xu W.-W. (2024). Facilitated the Discovery of New γ/Γ′ Co-Based Superalloys by Combining First-Principles and Machine Learning. npj Comput. Mater..

[204] Xiong J., Bai B.-W., Jiang H.-R., Faus-Golfe A. (2024). Determinants of Saturation Magnetic Flux Density in Fe-Based Metallic Glasses: Insights from Machine-Learning Models. Rare Met..

[205] Li P., Yang Y., Chen C. (2024). Research on Fatigue Crack Propagation Prediction for Marine Structures Based on Automated Machine Learning. J. Mar. Sci. Eng..

[206] Shin D., Yamamoto Y., Brady M.P., Lee S., Haynes J.A. (2019). Modern Data Analytics Approach to Predict Creep of High-Temperature Alloys. Acta Mater..

[207] Badini S., Regondi S., Pugliese R. (2023). Unleashing the Power of Artificial Intelligence in Materials Design. Materials.

[208] Zhang Y. (2024). Thoughts on Experimental Design of Materials Research in the Era of Artificial Intelligence. Adv. Ceram..

[209] Xiong J., Zhang T., Shi S. (2020). Machine Learning of Mechanical Properties of Steels. Sci. China Technol. Sci..

[210] Mamun O., Wenzlick M., Hawk J., Devanathan R. (2021). A Machine Learning Aided Interpretable Model for Rupture Strength Prediction in Fe-Based Martensitic and Austenitic Alloys. Sci. Rep..

[211] Roy A., Taufique M.F.N., Khakurel H., Devanathan R., Johnson D.D., Balasubramanian G. (2022). Machine-Learning-Guided Descriptor Selection for Predicting Corrosion Resistance in Multi-Principal Element Alloys. npj Mater. Degrad..

[212] Roy A., Hussain A., Sharma P., Balasubramanian G., Taufique M.F.N., Devanathan R., Singh P., Johnson D.D. (2023). Rapid Discovery of High Hardness Multi-Principal-Element Alloys Using a Generative Adversarial Network Model. Acta Mater..

[213] Tang Y., Wan Y., Wang Z., Zhang C., Han J., Hu C., Tang C. (2022). Machine Learning and Python Assisted Design and Verification of Fe–Based Amorphous/Nanocrystalline Alloy. Mater. Des..

[214] Liu Z., Zhang L., Liu C., Tan K., Ma A., Zheng Y. (2024). Understanding of Tribocorrosion and Corrosion Characteristics of 304L Stainless Steel in Hot Concentrated Nitric Acid Solution. J. Cent. South Univ..

[215] Chen F., Zhou J., Su C., Zhang C., Zhang H., Guo S. (2024). Catalytic Properties of Fe-Based Amorphous Alloys with Different Mo Content after Acid Corrosion. J. Non-Cryst. Solids.

[216] Gao F., Gui F., Feng D., Qu X., Hu F., Yang X. (2023). Study on the Corrosion Behavior of D36 Steel Plate and H62 Copper Alloy Net for Marine Aquaculture Facilities in Simulated Seawater. J. Mar. Sci. Eng..

[217] Gao Y., Wu J., Zhang D., Wang P., Wang Y., Li C., Zhu L., Wang W., Zheng R., Sun C. (2024). Corrosion of Q235 Steel Affected by Pseudodesulfovibrio Cashew Differed with Electron Acceptors. J. Cent. South Univ..

[218] Zhao Y.-C., Ma H.-W., Sun J.-D., Luo J.-H., Su Y., Feng L., Liu T.-Z., Zhan F.-Q., Yu Z.-Q., Yang T. (2025). A Xanthium Sibiricum Biomimetic Fe-Based Medium-Entropy Alloy with Significant Antibacterial and Mechanical Behaviors. Rare Met..

[219] Qiu X., Fan X., Xu H., Li L., Jiang H., Chen C. (2023). Corrosion Characteristics of Low-Carbon Steel Anchor Bolts in a Carbonaceous Mudstone Environment. J. Cent. South Univ..

[220] Zhou X., Wang Z., Ou-Yang Z., Su H., Wang Y., Li Z., Wu T. (2024). Effect of Cathode Protection on Desulfovibrio Desulfuricans Corrosion of X80 Steel in a Marine Tidal Environment. J. Cent. South Univ..

[221] Jianian S., Longjiang Z., Tiefan L. (1997). High-Temperature Oxidation of Fe-Cr Alloys in Wet Oxygen. Oxid. Met..

[222] McKamey C.G., DeVan J.H., Tortorelli P.F., Sikka V.K. (1991). A Review of Recent Developments in Fe_3_ Al-Based Alloys. J. Mater. Res..

[223] Kumar R., Singh Raman R.K., Bakshi S.R., Raja V.S., Parida S. (2024). Exploring the Influence of Nanocrystalline Structure and Aluminum Content on High-Temperature Oxidation Behavior of Fe-Cr-Al Alloys. Materials.

[224] Elliott P. Practical Guide to High-Temperature Alloys (10056). https://nickelinstitute.org/en/library/technical-guides/practical-guide-to-high-temperature-alloys-10056/.

[225] Wood G.C. (1970). High-Temperature Oxidation of Alloys. Oxid. Met..

[226] Deevi S.C., Sikka V.K. (1996). Nickel and Iron Aluminides: An Overview on Properties, Processing, and Applications. Intermetallics.

[227] Yu R.H., Basu S., Ren L., Zhang Y., Parvizi-Majidi A., Unruh K.M., Xiao J.Q. (2000). High Temperature Soft Magnetic Materials: FeCo Alloys and Composites. IEEE Trans. Magn..

[228] Luo W., Hou T., Liang X., Zhang D., Lin H., Li Y., Zhao T., Hu C., Yershov S., Wu K. (2024). Electronic-Scale Assessment of High-Temperature Oxidation Mechanisms in a Novel Fe-Based Alloy. J. Iron Steel Res. Int..

[229] Becker L., Radtke F., Lentz J., Benito S., Broeckmann C., Weber S. (2024). Powder Metallurgy and Additive Manufacturing of High-Nitrogen Alloyed FeCr(Si)N Stainless Steel. Adv. Eng. Mater..

[230] Wieczerzak K., Stygar M., Brylewski T., Chulist R., Bała P., Michler J. (2024). Kinetics and Mechanisms of High-Temperature Oxidation in BCC and FCC High-Alloy Fe-Based Alloys with High Volume Fraction of Carbides. Mater. Des..

[231] He M.-R., Wang S., Shi S., Jin K., Bei H., Yasuda K., Matsumura S., Higashida K., Robertson I.M. (2017). Mechanisms of Radiation-Induced Segregation in CrFeCoNi-Based Single-Phase Concentrated Solid Solution Alloys. Acta Mater..

[232] El-Atwani O., Nathaniel J.E., Leff A.C., Baldwin J.K., Hattar K., Taheri M.L. (2017). Evidence of a Temperature Transition for Denuded Zone Formation in Nanocrystalline Fe under He Irradiation. Mater. Res. Lett..

[233] Yang T., Guo W., Poplawsky J.D., Li D., Wang L., Li Y., Hu W., Crespillo M.L., Yan Z., Zhang Y. (2020). Structural Damage and Phase Stability of Al0.3CoCrFeNi High Entropy Alloy under High Temperature Ion Irradiation. Acta Mater..

[234] Jin H.-H., Ko E., Lim S., Kwon J., Shin C. (2017). Effect of Irradiation Temperature on Microstructural Changes in Self-Ion Irradiated Austenitic Stainless Steel. J. Nucl. Mater..

[235] Hussain A., Dhaka R.S., Ryu H.J., Sharma S.K., Kulriya P.K. (2023). A Critical Review on Temperature Dependent Irradiation Response of High Entropy Alloys. J. Alloys Compd..

[236] Wu W., Yang Q., Zhang W., Zhang Y., Yan D., Li Z. (2025). Insights into Irradiation-Induced Defect Evolution and Segregation in Metastable High-Entropy Alloys: Effects of High-Density Incoherent Planar Defects and Temperature. Acta Mater..

[237] Liu S., Zhang J., Wang H., Liu G., Ding X., Sun J. (2024). Designing Ultrastrong and Thermally Stable FeCrAl Alloys with the Fine-Grained Structure. J. Mater. Sci. Technol..

[238] Wang X., Shen X. (2023). Research Progress of ODS FeCrAl Alloys–A Review of Composition Design. Materials.

[239] Gong X., Li R., Sun M., Ren Q., Liu T., Short M.P. (2016). Opportunities for the LWR ATF Materials Development Program to Contribute to the LBE-Cooled ADS Materials Qualification Program. J. Nucl. Mater..

[240] Cheng Z., Sun J., Gao X., Wang Y., Cui J., Wang T., Chang H. (2023). Irradiation Effects in High-Entropy Alloys and Their Applications. J. Alloys Compd..

[241] Tan F., Li L., Li J., Liu B., Liaw P.K., Fang Q. (2023). Multiscale Modelling of Irradiation Damage Behavior in High Entropy Alloys. Adv. Powder Mater..

[242] Ostovari Moghaddam A., Cabot A., Trofimov E.A. (2021). Does the Pathway for Development of next Generation Nuclear Materials Straightly Go through High-Entropy Materials?. Int. J. Refract. Met. Hard Mater..

[243] Bigdeli S., Kjellqvist L., Naraghi R., Höglund L., Larsson H., Jonsson T. (2021). Strategies for High-Temperature Corrosion Simulations of Fe-Based Alloys Using the Calphad Approach: Part I. J. Phase Equilibria Diffus..

[244] Lu J., Ren G., Chen Y., Zhang H., Li L., Huang A., Liu X., Cai H., Shan X., Luo L. (2022). Unraveling the Oxidation Mechanism of an AlCoCrFeNi High-Entropy Alloy at 1100 °C. Corros. Sci..

[245] Sun Z., Bei H., Yamamoto Y. (2017). Microstructural Control of FeCrAl Alloys Using Mo and Nb Additions. Mater. Charact..

[246] Niu B., Wang Z., Wang Q., Pan Q., Dong C., Zhang R., Liu H., Liaw P.K., Xu W. (2020). Dual-Phase Synergetic Precipitation in Nb/Ta/Zr Co-Modified Fe–Cr–Al–Mo Alloy. Intermetallics.

[247] Wen D., Jiang B., Huang Z., Kong F., Jiang X., Wang A., Hou X., Luan J., Wang Q., Liu G. (2022). Rational Design and Multi-Stage Formation Mechanisms of FeCrNiAl Medium-Entropy Alloy Strengthened by Multi-Scaled Dual Phases. Mater. Charact..

[248] Niu M.C., Qiu S., Yu Q., Li W., Zhang S.Z., Guo J.M., Luan J.H., Wang W., Yang T., Wang X.L. (2025). Achieving Excellent Elevated-Temperature Mechanical Properties in Dual-Phase High-Entropy Alloys via Nanoscale Co-Precipitation and Heterostructure Engineering. Acta Mater..

[249] Liu L., Zhang Y., Han J., Wang X., Jiang W., Liu C., Zhang Z., Liaw P.K. (2021). Nanoprecipitate-Strengthened High-Entropy Alloys. Adv. Sci..

[250] Sun D., Lu Z., Li H., Li X., Gao S., Xian J., Jin F. (2025). Strength-Ductility Synergy of ODS-FeCrAl Alloy by Introduction of High-Entropy Reinforcements with Different Al Contents. J. Alloys Compd..

[251] Deng L., Wang C., Luo J., Tu J., Guo N., Xu H., He P., Xia S., Yao Z. (2022). Preparation and Property Optimization of FeCrAl-Based ODS Alloy by Machine Learning Combined with Wedge-Shaped Hot-Rolling. Mater. Charact..

[252] Nair A., Kumanan S., Prakash C., Mohan D.G., Saxena K.K., Kumar S., Kumar G. (2023). Research Developments and Technological Advancements in Conventional and Non-Conventional Machining of Superalloys—A Review. J. Adhes. Sci. Technol..

[253] Meng Q., Lai L., Rao W., Li A., Yu H., La P. (2024). Creep Properties and Corrosion Behavior of TP347H Stainless Steel with Al in Molten Carbonate Salt. Materials.

[254] Felix-Contreras R., De La Vega Olivas J., Arrieta-Gonzalez C.D., Chacon-Nava J.G., Rodriguez-Diaz R.A., Gonzalez-Rodriguez J.G., Porcayo-Calderon J. (2024). Kinetic and Thermodynamic Aspects of the Degradation of Ferritic Steels Immersed in Solar Salt. Materials.

[255] Lee U., Kim M.W., Na J., Lee M., Kim S.J., Kim D.-J., Yoon Y.S. (2025). A Study on the Corrosion Behavior of Fe/Ni-Based Structural Materials in Unpurified Molten Chloride Salt. Materials.

[256] Du K., Gao E., Zhang C., Ma Y., Wang P., Yu R., Li W., Zheng K., Cheng X., Tang D. (2023). An Iron-Base Oxygen-Evolution Electrode for High-Temperature Electrolyzers. Nat. Commun..

[257] Tang C., Shi H., Jianu A., Weisenburger A., Victor G., Grosse M., Müller G., Seifert H.J., Steinbrück M. (2021). High-Temperature Oxidation of AlCrFeNi-(Mn or Co) High-Entropy Alloys: Effect of Atmosphere and Reactive Element Addition. Corros. Sci..

[258] Zhang X., Xu C., Chen Y., Chen W.-Y., Park J.-S., Kenesei P., Almer J., Burns J., Wu Y., Li M. (2020). High-Energy Synchrotron x-Ray Study of Deformation-Induced Martensitic Transformation in a Neutron-Irradiated Type 316 Stainless Steel. Acta Mater..

[259] Kotan H., Darling K.A., Luckenbaugh T. (2021). High Temperature Mechanical Properties and Microstructures of Thermally Stabilized Fe-Based Alloys Synthesized by Mechanical Alloying Followed by Hot Extrusion. Met. Mater. Int..

[260] Shi C., Zhu X., Zheng X., Lan P., Li J. (2022). Precipitation and Growth of Laves Phase and NbC during Aging and Its Effect on Tensile Properties of a Novel 15Cr–22Ni–1Nb Austenitic Heat-Resistant Steel. Mater. Sci. Eng. A.

[261] Stepanov N.D., Shaysultanov D.G., Tikhonovsky M.A., Zherebtsov S.V. (2018). Structure and High Temperature Mechanical Properties of Novel Non-Equiatomic Fe-(Co, Mn)-Cr-Ni-Al-(Ti) High Entropy Alloys. Intermetallics.

[262] Carsí M., Llaneza J., Ruano O.A. (2019). Microstructure and Stability Conditions for Hot Deformation of a Modified Iron-Based Superalloy. Mater. Sci. Technol..

[263] Ji G., Li F., Li Q., Li H., Li Z. (2011). A Comparative Study on Arrhenius-Type Constitutive Model and Artificial Neural Network Model to Predict High-Temperature Deformation Behaviour in Aermet100 Steel. Mater. Sci. Eng. A.

[264] Xu T., Li J., Yu Y., Li T., Wang L., Tang H., Qiao Z. (2025). High-Temperature Tribological Properties of Fe50Mn25Cr5Al15Ti5 Iron-Based High-Entropy Alloys. Tribol. Int..

[265] Wakai E., Noto H., Shibayama T., Furuya K., Ando M., Kamada T., Ishida T., Makimura S. (2024). Microstructures and Hardness of BCC Phase Iron-Based High Entropy Alloy Fe-Mn-Cr-V-Al-C. Mater. Charact..

[266] Winkelmann H., Varga M., Badisch E., Danninger H. (2009). Wear Mechanisms at High Temperatures: Part 2: Temperature Effect on Wear Mechanisms in the Erosion Test. Tribol. Lett..

[267] Singla Y.K., Maughan M.R., Arora N., Dwivedi D.K. (2024). Enhancing the Wear Resistance of Iron-Based Alloys: A Comprehensive Review of Alloying Element Effects. J. Manuf. Process..

[268] Vo N.Q., Liebscher C.H., Rawlings M.J.S., Asta M., Dunand D.C. (2014). Creep Properties and Microstructure of a Precipitation-Strengthened Ferritic Fe–Al–Ni–Cr Alloy. Acta Mater..

[269] Rawlings M.J.S., Liebscher C.H., Asta M., Dunand D.C. (2017). Effect of Titanium Additions upon Microstructure and Properties of Precipitation-Strengthened Fe-Ni-Al-Cr Ferritic Alloys. Acta Mater..

[270] Baik S.-I., Rawlings M.J.S., Dunand D.C. (2018). Effect of Hafnium Micro-Addition on Precipitate Microstructure and Creep Properties of a Fe-Ni-Al-Cr-Ti Ferritic Superalloy. Acta Mater..

[271] Dymáček P., Jarý M., Bártková D., Luptáková N., Gamanov Š., Bořil P., Georgiev V., Svoboda J. (2024). High-Temperature Creep Resistance of FeAlOY ODS Ferritic Alloy. Materials.

[272] Lu Q., Van Der Zwaag S., Xu W. (2016). High-Throughput Design of Low-Activation, High-Strength Creep-Resistant Steels for Nuclear-Reactor Applications. J. Nucl. Mater..

[273] AlMangour B., Baek M.-S., Grzesiak D., Lee K.-A. (2018). Strengthening of Stainless Steel by Titanium Carbide Addition and Grain Refinement during Selective Laser Melting. Mater. Sci. Eng. A.

[274] AlMangour B., Kim Y.-K., Grzesiak D., Lee K.-A. (2019). Novel TiB2-Reinforced 316L Stainless Steel Nanocomposites with Excellent Room- and High-Temperature Yield Strength Developed by Additive Manufacturing. Compos. Part. B Eng..

[275] Han J.Y., Wang L., Hu P., Hu B.L., Ma S.J., Gao L.L., Bai R., Wang Q., Feng R., Jin B. (2024). Research Progress in Modification of MoSi_2_ Coatings on Surface of Refractory Metals and Their Alloys: A Review. Rare Met..

[276] Shunan N., Lijun Z., Xudong L., Dianli Q., Jiaao P. (2024). Research Progress of Al2O3-SiC Composite Ceramic. Adv. Ceram..

[277] Liao M., Hu X., Zhong C., Xu P., Wang X., Zhang Z., Zhou P., Zhang M., Su Z., Huang Q. (2024). Controlling the Si/C Ratio in SiC Matrix Based on the Modified Polymethysilane for C/C–SiC Composites with Enhanced Mechanical Properties. J. Adv. Ceram..

[278] Miracle D.B. (2005). Metal Matrix Composites—From Science to Technological Significance. Compos. Sci. Technol..

[279] Shi C., Liu X., Hao D., Lu H., Chen Y. (2024). Research on Interfacial Bonding Strength Between Alloy Matrix and PcBN Composite Sheet. Adv. Ceram..

[280] Dong W., Zhao Y., Wang X., Yuan X., Bu K., Dong C., Wang R., Huang F. (2018). Boron Embedded in Metal Iron Matrix as a Novel Anode Material of Excellent Performance. Adv. Mater..

[281] Moureaux F., Stevens P., Toussaint G., Chatenet M. (2013). Development of an Oxygen-Evolution Electrode from 316L Stainless Steel: Application to the Oxygen Evolution Reaction in Aqueous Lithium–Air Batteries. J. Power Sources.

[282] Moureaux F., Stevens P., Toussaint G., Chatenet M. (2019). Timely-Activated 316L Stainless Steel: A Low Cost, Durable and Active Electrode for Oxygen Evolution Reaction in Concentrated Alkaline Environments. Appl. Catal. B Environ..

[283] Okonkwo P.C., Barhoumi E.M., Ben Belgacem I., Mansir I.B., Aliyu M., Emori W., Uzoma P.C., Beitelmal W.H., Akyüz E., Radwan A.B. (2023). A Focused Review of the Hydrogen Storage Tank Embrittlement Mechanism Process. Int. J. Hydrogen Energy.

[284] Dwivedi S.K., Vishwakarma M. (2018). Hydrogen Embrittlement in Different Materials: A Review. Int. J. Hydrogen Energy.

[285] Huang L., Chen D., Xie D., Li S., Zhang Y., Zhu T., Raabe D., Ma E., Li J., Shan Z. (2023). Quantitative Tests Revealing Hydrogen-Enhanced Dislocation Motion in α-Iron. Nat. Mater..

[286] Luo H., Pan Z., Yang T., Chang W., Zhang D., Cheng H., Li X., Raabe D. (2025). A High-Entropy Alloy for Superior Resistance to Biogenic Sulfuric Acid Corrosion and Hydrogen Embrittlement. Matter.

[287] Ustolin F., Paltrinieri N., Berto F. (2020). Loss of Integrity of Hydrogen Technologies: A Critical Review. Int. J. Hydrogen Energy.

[288] Briottet L., Batisse R., Bernard P., Duret-Thual C., Heuzé J.-L., Martin F., Thebault F., Vucko F. (2019). Industrial Consequences of Hydrogen Embrittlement. Mechanics—Microstructure—Corrosion Coupling.

[289] Balitskii A.I., Ivaskevich L.M. (2018). Assessment of Hydrogen Embrittlement in High-Alloy Chromium-Nickel Steels and Alloys in Hydrogen at High Pressures and Temperatures. Strength Mater..

[290] Gong P., Turk A., Nutter J., Yu F., Wynne B., Rivera-Diaz-del-Castillo P., Mark Rainforth W. (2022). Hydrogen Embrittlement Mechanisms in Advanced High Strength Steel. Acta Mater..

[291] Kwon Y.J., Lee T., Lee J., Chun Y.S., Lee C.S. (2015). Role of Cu on Hydrogen Embrittlement Behavior in Fe–Mn–C–Cu TWIP Steel. Int. J. Hydrogen Energy.

[292] Koyama M., Akiyama E., Lee Y.-K., Raabe D., Tsuzaki K. (2017). Overview of Hydrogen Embrittlement in High-Mn Steels. Int. J. Hydrogen Energy.

[293] Li X., Yin J., Zhang J., Wang Y., Song X., Zhang Y., Ren X. (2022). Hydrogen Embrittlement and Failure Mechanisms of Multi-Principal Element Alloys: A Review. J. Mater. Sci. Technol..

[294] Tan S.M., Gao S.J., Wan X.J. (1993). Temperature Effects on Gaseous Hydrogen Embrittlement of a High-Strength Steel. J. Mater. Sci. Lett..

[295] Okuno K., Takai K. (2023). Determination of Hydrogen Diffusibility and Embrittlement Susceptibility of High-Strength Steel Evaluated at Different Temperatures Based on the Local Equilibrium Theory. Acta Mater..

[296] Neuharth J.J., Cavalli M.N. (2015). Investigation of High-Temperature Hydrogen Embrittlement of Sensitized Austenitic Stainless Steels. Eng. Fail. Anal..

[297] Savaedi Z., Motallebi R., Mirzadeh H., Malekan M. (2022). Superplasticity of Bulk Metallic Glasses (BMGs): A Review. J. Non-Cryst. Solids.

[298] Jiang L., Bao M., Dong Y., Yuan Y., Zhou X., Meng X. (2023). Processing, Production and Anticorrosion Behavior of Metallic Glasses: A Critical Review. J. Non-Cryst. Solids.

[299] Lashgari H.R., Chu D., Xie S., Sun H., Ferry M., Li S. (2014). Composition Dependence of the Microstructure and Soft Magnetic Properties of Fe-Based Amorphous/Nanocrystalline Alloys: A Review Study. J. Non-Cryst. Solids.

[300] Liu T., Li F., Wang A., Xie L., He Q.F., Luan J., He A., Wang X., Liu C.T., Yang Y. (2019). High Performance Fe-Based Nanocrystalline Alloys with Excellent Thermal Stability. J. Alloys Compd..

[301] Wei J., Zheng Z., Qiu Z., Zeng D. (2025). Insight into Efficient Removal of Tetracycline from Water by Fe–Si–B Amorphous Alloys. J. Non-Cryst. Solids.

[302] Li H., Wang A., Liu T., Chen P., He A., Li Q., Luan J., Liu C.T. (2021). Design of Fe-Based Nanocrystalline Alloys with Superior Magnetization and Manufacturability. Mater. Today.

[303] Inoue A., Shen B.L. (2004). A New Fe-Based Bulk Glassy Alloy with Outstanding Mechanical Properties. Adv. Mater..

[304] Xu F., Yao H., Tang K., Li Y., Han F., Tan Z., He D., Yang Y., Liu Y., Zhou Z. (2023). Degeneration of Thermal Insulation Property for Fe-Based Amorphous Coating during Long-Term Heat Exposure. J. Non-Cryst. Solids.

[305] Knipling K.E., Daniil M., Willard M.A. (2009). Fe-Based Nanocrystalline Soft Magnetic Alloys for High-Temperature Applications. Appl. Phys. Lett..

[306] Li Y., Jia X., Zhang W., Fang C., Wang X., Qin F., Yamaura S., Yokoyama Y. (2014). Effects of Alloying Elements on the Thermal Stability and Corrosion Resistance of an Fe-Based Metallic Glass with Low Glass Transition Temperature. Met. Mater. Trans. A.

[307] Ding J., Xu H., Shi Z., Li X., Zhang T. (2021). Effect of Primary α-Fe on Soft Magnetic Properties of FeCuNbSiB Amorphous/Nanocrystalline Alloy. J. Non-Cryst. Solids.

[308] Hou L., Li M., Jiang C., Fan X., Luo Q., Chen S., Song P., Li W. (2021). Thermal and Magnetic Properties of Fe(Co)BCCu Amorphous Alloys with High Saturation Magnetization of 1.77 T. J. Alloys Compd..

[309] Shi L., Hu X., Li Y., Yuan G., Yao K. (2021). The Complementary Effects of Fe and Metalloids on the Saturation Magnetization of Fe-Based Amorphous Alloys. Intermetallics.

[310] Yang Z.Z., Zhu L., Ye L.X., Gao X., Jiang S.S., Yang H., Wang Y.G. (2021). Nanoscale Structural Heterogeneity Perspective on the Improved Magnetic Properties during Relaxation in a Fe-Based Metallic Glass. J. Non-Cryst. Solids.

[311] Meng Y., Pang S., Chang C., Bai X., Zhang T. (2021). Nanocrystalline Fe83Si4B10P2Cu1 Ribbons with Improved Soft Magnetic Properties and Bendability Prepared via Rapid Annealing of the Amorphous Precursor. J. Magn. Magn. Mater..

[312] Diaz Y., Sevilla A., Mónaco A., Méndez F.J., Rosales P., García L., Brito J.L. (2013). Metallic Monoliths of AISI 304 Stainless Steel, Aluminum, FeCrAlloy^®^ and Brass, Coated by Mo and W Oxides for Thiophene Hydrodesulfurization. Fuel.

[313] Magnier L., Cossard G., Martin V., Pascal C., Roche V., Sibert E., Shchedrina I., Bousquet R., Parry V., Chatenet M. (2024). Fe–Ni-Based Alloys as Highly Active and Low-Cost Oxygen Evolution Reaction Catalyst in Alkaline Media. Nat. Mater..

[314] Li H., Lei F., Li Z., Li G., Zhao Y. (2023). Research Progress of Iron-Based Oxygen Reduction Catalysts in Fuel Cells. Fine Chem..

[315] Jia Z., Wang J.C., Liang S.X., Zhang W.C., Wang W.M., Zhang L.C. (2017). Activation of Peroxymonosulfate by Fe78Si9B13 Metallic Glass: The Influence of Crystallization. J. Alloys Compd..

[316] Li Y., Liu Y., Zhan Y., Zhang Y., Zhao X., Yang M., Ruan W., Zhang Z., Liang X., Ma J. (2024). Peracetic Acid-Induced Nanoengineering of Fe-Based Metallic Glass Ribbon in Application of Efficient Drinking Water Treatment. Appl. Catal. B Environ. Energy.

[317] Zhang C., Sun Q. (2017). Annealing-Induced Different Decolorization Performances of Fe-Mo-Si-B Amorphous Alloys. J. Non-Cryst. Solids.

[318] Chen S., Chen N., Cheng M., Luo S., Shao Y., Yao K. (2017). Multi-Phase Nanocrystallization Induced Fast Degradation of Methyl Orange by Annealing Fe-Based Amorphous Ribbons. Intermetallics.

[319] Zhang C., Zhu Z., Zhang H. (2017). Effects of the Addition of Co, Ni or Cr on the Decolorization Properties of Fe-Si-B Amorphous Alloys. J. Phys. Chem. Solids.

[320] Wang C., Tonna C., Mei D., Buhagiar J., Zheludkevich M.L., Lamaka S.V. (2022). Biodegradation Behaviour of Fe-Based Alloys in Hanks’ Balanced Salt Solutions: Part II. The Evolution of Local pH and Dissolved Oxygen Concentration at Metal Interface. Bioact. Mater..

[321] Liu S., Guo H. (2024). A Short Review of Antibacterial Cu-Bearing Stainless Steel: Antibacterial Mechanisms, Corrosion Resistance, and Novel Preparation Techniques. J. Iron Steel Res. Int..

[322] Dong C., Xu Q., Ma Y. (2024). Towards High-Field Applications: High-Performance, Low-Cost Iron-Based Superconductors. Natl. Sci. Rev..

[323] Paglione J., Greene R.L. (2010). High-Temperature Superconductivity in Iron-Based Materials. Nat. Phys..

[324] Kim A.S., Camp F.E. (1996). High Performance NdFeB Magnets (Invited). J. Appl. Phys..

[325] Crozier-Bioud T., Momeni V., Gonzalez-Gutierrez J., Kukla C., Luca S., Rolere S. (2023). Current Challenges in NdFeB Permanent Magnets Manufacturing by Powder Injection Molding (PIM): A Review. Mater. Today Phys..

[326] Sundar R.S., Deevi S.C. (2005). Soft Magnetic FeCo Alloys: Alloy Development, Processing, and Properties. Int. Mater. Rev..

[327] Fei Y., Yao J., Cheng W., Jiao W. (2024). Hierarchical 3D FeCoNi Alloy/CNT @ Carbon Nanofiber Sponges as High-Performance Microwave Absorbers with Infrared Camouflage. Materials.

[328] Han Y., Zhang X., Wei T., Zhan J. (2022). Hydrothermal Preparation of Iron/Carbon Composites and Their Degradation for Trichloroethylene in Groundwater. Fine Chem..

